# Histone Demethylase Jumonji D3 (*JMJD3*) as a Tumor Suppressor by Regulating p53 Protein Nuclear Stabilization

**DOI:** 10.1371/journal.pone.0051407

**Published:** 2012-12-07

**Authors:** Chibawanye I. Ene, Lincoln Edwards, Gregory Riddick, Mehmet Baysan, Kevin Woolard, Svetlana Kotliarova, Chen Lai, Galina Belova, Maggie Cam, Jennifer Walling, Ming Zhou, Holly Stevenson, Hong Sug Kim, Keith Killian, Timothy Veenstra, Rolanda Bailey, Hua Song, Wei Zhang, Howard A. Fine

**Affiliations:** 1 Neuro-Oncology Branch, Center for Cancer Research, National Cancer Institute, National Institute of Neurological Disorders and Stroke, National Institutes of Health, Bethesda, Maryland, United States of America; 2 NIH-Oxford-Cambridge Research Scholars Program, National Institute of Health (NIH), Bethesda, Maryland, United States of America; 3 Laboratory of Proteomics and Analytical Technologies, Frederick National Laboratory for Cancer Research, National Cancer Institute, National Institute of Health (NIH), Frederick, Maryland, United States of America; 4 Molecular Profiling Core, Genetics Branch, National Cancer Institute, National Institute of Health (NIH), Bethesda, Maryland, United States of America; 5 New York University Cancer Center, New York University, Langone Medical Center, New York, New York, United States of America; Peking University Health Science Center, China

## Abstract

Histone methylation regulates normal stem cell fate decisions through a coordinated interplay between histone methyltransferases and demethylases at lineage specific genes. Malignant transformation is associated with aberrant accumulation of repressive histone modifications, such as polycomb mediated histone 3 lysine 27 (H3K27me3) resulting in a histone methylation mediated block to differentiation**.** The relevance, however, of histone demethylases in cancer remains less clear. We report that *JMJD3,* a H3K27me3 demethylase, is induced during differentiation of glioblastoma stem cells (GSCs), where it promotes a differentiation-like phenotype via chromatin dependent (*INK4A/ARF* locus activation) and chromatin independent (nuclear p53 protein stabilization) mechanisms. Our findings indicate that deregulation of JMJD3 may contribute to gliomagenesis via inhibition of the p53 pathway resulting in a block to terminal differentiation.

## Introduction

Recent evidence suggests that histone methylation is a dynamic process that modulates transcriptional activity in both normal and cancer cells[1]–[6]. Histone methylation is a result of a coordinated interplay between histone methyltransferases and demethylases at lineage specific gene promoters thereby contributing to normal development by regulating cell fate decisions [4], [7]. The relevance of the interplay between histone methyltransferases and demethylases during differentiation has been demonstrated in the developing central nervous system [8], [9]. Here, it was shown that polycomb protein deletion in embryonic neural stem cells (NSCs) accelerates either neurogenesis or gliogenesis depending on the polycomb component deleted and timing of deletion [8]–[10]. Moreover, others showed that the H3K27 demethylase JMJD3 is required for re-activation of neuronal specific genes during differentiation of embryonic NSCs [4]. In these experiments it was shown that the retinoic acid co-repressor NCOR-2 suppresses JMJD3 expression. Retinoic acid treatment destabilizes nuclear NCOR-2 resulting in de-repression of the *JMJD3* promoter and JMJD3 mediated induction of neurogenic differentiation. Moreover, NCOR-2 mediated transcriptional suppression was also previously shown to inhibit astrocytic differentiation [11]. Altogether, these results suggest that a coordinated temporal interplay between EZH2 and JMJD3 modulate stem cell self-renewal and lineage specific gene expression of NSCs.

It was been demonstrated that primary human glioblastomas have stem-like cells, termed glioblastoma stem cells (GSCs), that can be enriched for using the neural stem cell marker CD133 and CD15 [12], [13]. GSCs isolated from primary glioblastoma patient samples maintain the genotype and phenotype of the original tumour sample from which they were derived [14], [15]. GSCs also maintained genomic stability for up to 35 passages *in vitro* when they are cultured in neural stem cell media containing epidermal growth factor (EGF) and fibroblast growth factor (FGF). Culturing GSCs in serum containing media such as standard immortalized glioma cell line media (*DMEM* with 10% foetal bovine serum), however, results in genomic instability, aneuploidy and significantly different gene expression profiles compared to the parental glioblastoma tumors from which they were derived [14]. Furthermore, GSCs more accurately recapitulate clinical aspects of human glioblastomas such as white matter migration/invasion, vascular proliferation, necrosis and cellular heterogeneity, features that are absent in immortalized glioma cell lines such as U87 and U251 [14]. Therefore, GSCs are a more representative model of the actual patient disease compared to the long established immortalized glioma cell lines.

In astrocytes, malignant transformation is associated with aberrant accumulation of repressive histone modifications, such as tri-methylated histone 3 lysine 27 (H3K27me3) mediated by histone methyltransferases such as EZH2, resulting in a histone methylation mediated block to differentiation [1], [16]. The relevance, however, of histone demethylases in cancer remains less clear. We report that *JMJD3,* a H3K27me3 specific demethylase, is induced during differentiation of patient-derived glioblastoma stem cells (GSCs), where it promotes a differentiation-like phenotype via a chromatin dependent (*INK4A/ARF* locus activation) and chromatin independent (direct p53 protein stabilization) mechanism resulting in a p53-mediated cell cycle arrest and differentiation. We demonstrate that a subset of GBMs have somatic mutations of *JMJD3* or down regulation of its mRNA expression secondary to DNA hypermethylation of an intragenic regulatory element. JMJD3 re-activation via regulatory element demethylation results in p53-mediated differentiation of GSCs and suppression of tumorgenicity. Our findings demonstrate a tumor suppressor function for a histone demethylase and indicate that deregulation of JMJD3 either through somatic mutations, epigenetic repression of its mRNA expression or NCoR2 mediated repression may contribute to human gliomagenesis via inhibition of the p53 pathway resulting in a block to terminal differentiation.

## Methods

### Differentiation of Glioblastoma Stem Cells (GSCs)

After written consent tumor samples were obtained from patients undergoing surgery at the National Institutes of Health (NIH) in accordance with the surgical procedures of the National Cancer Institute's Institutional Review Board that specifically approved this study. Tissue samples were enzymatically dissociated and cultured as previously described. 6- well plates were coated with poly-ornithine (Sigma P4957) for 1 hr at 37°C and washed 3 times with PBS. Cells were plated at 5E^5^ cells per well (6-well plate) and treated with 2 µM all trans-retinoic acid, RA (Sigma R2625) for 5 days. Media was changed approximately every 2–3 days to maintain a constant dose of RA. On day 5, RNA was collected using Qiagen RNeasy Kit (74106). Cells were lysed directly on the 6-well plates and total RNA was isolated following the manufacturers protocol. All experiments were done in triplicates. Error bars represent means ±SD.

### Quantitative Real Time PCR (RT-qPCR)

500 ng of high-quality RNA from the RA treatments was reversed transcribed using the applied biosystems taqman reverse transcription reagents (N8080234) according to manufacturer’s protocol. RT-qPCR was performed using reagents from applied biosystems taqman universal PCR mix (4304437) and 20 nM of FAM- tagged gene specific primers on the 7900HT real time PCR system. Primers from applied biosystems were as follows: human *p16/p14* (Primers span *INK4A* and *ARF* shared Exon 2-Exon 3; Hs00923894_m1), human and mouse *GFAP* (Hs 00909236_m1, Mm01253033_m1), human *p21* (Hs 00355782_m1), human and mouse *JMJD3* (Primers span Exon1-Exon2; Hs 00389738_m1, Mm 01332680_m1), human *TP53* (Hs00153349_m1). Relative gene expression was determined from the difference in Ct values between specific genes and endogenous *GAPDH* control (ΔCt). Fold change is represented as 2^−ΔΔCt^. All experiments were done in triplicates. Error bars represent means ±SD.

### JMJD3 Functional Studies

For transient JMJD3 overexpression, 4E^6^ GSCs were nucleofected with 4 µg of empty p3xFLAG-CMV expression vector (Sigma), p3xFLAG-CMV-JMJD3 wild-type (JMJD3) or p3xFLAG-CMV-JMJD3 mutant (JMJD3 MT) using the mouse neural stem cell nucleofector kit by Amaxa (VPG-1004) according to manufacturer’s protocol. Lipofectamine 2000 (Invitrogen 11668-019) was used to transfect HEK 293 cells according to manufacturer’s protocol. Briefly, 7E^5^ cells were seeded in 6-well plates and allowed to adhere overnight. Transfections were done at a ratio of 1 µg DNA: 3 µl Lipofectamine 2000 per well. RNA and protein were isolated at 48 hr and 72 hr respectively. For siRNA knockdown, 200 nM JMJD3 *ON-TARGETplus SMARTpool* (Dharmafect L-023013-01) was transfected into GSCs using Dharmafect Duo transfection reagent (Dharmafect T-2010). For 5-Azacytidine (5-Aza; Sigma EA2385) treatment following JMJD3 siRNA transfection, JMJD3 siRNA were transfected twice within a 48 hr period, followed by 5-Aza (5 µM) treatment for 4 days.

### Luciferase Reporter Assay

The human *GFAP* promoter luciferase was from Albee Messing [17]. The episomal human *p21* promoter luciferase reporter _ENREF_41was from addgene (Plasmid 16451) courtesy of Bert Vogelstein [18]. A p53 binding site mutant *p21* promoter luciferase reporter was generated by site directed mutagenesis using QuickChange XL site directed mutagenesis kit (Stratagene 200516) with the following primers: 5′-*TTT CTG GCC GTC AGG AA* ***A**** A* *TC TCC CAA GAT TTT G*-3′ and 5′-*CAA AAT CTT GGG AGA T* ***T****T T* *CC TGA CGG CCA GAA A*-3′ targeting the distal 5′ *TP53* binding site targeting a site previously described to be critical for p53 activation of the *p21* promoter [19]. Transfections were done with 1 µg of JMJD3 expression construct, 1 µg of luciferase constructs and 100 ng of Renilla firefly luciferase construct pRL-TK (Promega E2241) for background normalization. Cells were lysed at 24–48 hr for luciferase assay using the promega dual luciferase reporter system (E1910) according to the manufacturer’s protocol. All experiments were done in triplicates. Error bars represent means ±SD.

### Nuclear Fractionation, Western Blotting and Immunocytochemistry

Nuclear fractionation was performed in HEK 293 cells (ATCC) and GSC 923 (Glioblastoma primary cell line derived from patient sample following the approval of National Cancer Institute Institutional Review Board) with the Proteoextract subcellular proteome extraction kit (Calbiochem 539790) according to the manufacturer’s protocol. For western analysis, 15–30 µg of protein lysate were run on a bis-tris 4–12% gradient gel (Invitrogen). Primary anti-bodies used were as follows: mouse anti-human p53 IC12 (Cell Signaling 2524) 1∶1000, mouse anti-Flag (Sigma F3165) 10 µg/ml, mouse anti-human p21 WAF/CIP1 12D1 (Cell Signaling 2947S) 1∶1000, rabbit anti-human GFAP (Dako M0761) 1∶4000, rabbit anti-human histone 3 (Millipore 04–928) 1∶250, mouse anti-human tubulin (Sigma T3526) 1∶5000, For immunocytochemistry, mouse anti-Flag-FITC Conjugated (Sigma F4049) 10 µg/ml, rabbit anti-human H3K27me3 (Millipore 07–449) 1∶500, monoclonal mouse anti-β-tubulin III 1∶1000 (Covance MMS-435P), polyclonal rabbit anti-β-tubulin III 1∶1000 (Convance PRB-435P) and rabbit anti-human GFAP (Dako M0761) 1∶4000.

### Co-immunoprecipitation (Co-IP)

Immunoprecipitations were performed in HEK 293 cells and GSC 923 according to the Santa Cruz Exacta-Cruz protocol (sc-450420 and sc-45056). Briefly, 500–1000 µg of protein lysate was incubated with mouse anti-IgG negative control ICO-97 (Santa Cruz sc 66186) 1∶50, mouse anti-Flag (Sigma F3165) 10 µg/ml and mouse anti-p53 IC12 (Cell Signaling 2524) 1∶500 or mouse anti-p53 DO1 (Santa Cruz sc-126). IP samples were analyzed on a bis-tris 4–12% gradient gel (Invitrogen) following Santa Cruz Exacta-Cruz protocol.

### Chromatin Immunoprecipitation (ChIP)

Chromatin immunoprecipitation was performed according to SABiosciences Champion CHIP-PCR manufacturer’s protocol (SABioscience 334471). Briefly HEK 293 cells grown in DMEM with 10% fetal bovine serum were transfected with either empty vector or JMJD3 using Lipofectamine 2000 (Invitrogen). Cells were cross-linked with 1% formaldehyde and the extracts were enzymatically digested as previously described [20]. Cross-linked material was immunoprecipitated with 4 µg of antibodies overnight at 4°C and followed by incubation with protein A beads (SABiosciences) for 1 hr at 4°C. After several washes, the complexes were eluted and the cross-linking was reversed according to SABiosciences protocol. Immunoprecipitated DNA was recovered by DNA extraction beads (SABiosciences) and analyzed by SYBR green real-time PCR. ΔCt values were determined by [Ct (target protein)- Ct (IgG negative control)]. Fold changes are represented as 2^−ΔΔCt^. The primers used were from SABioscience and corresponded to the *p21* transcription start sites (TSS), 1Kb upstream of TSS (−1Kb) and the region corresponding to the distal p53 binding site (−2 to −3Kb).

### Mass Spectrometry Analysis

To detect JMJD3 interacting proteins, empty vector (p3xFLAG-CMV, Sigma) and JMJD3-Flag were transfected into HEK293 cells using Lipofectamine 2000 (Invitrogen). 2 days following transfection, cells were lysed with 1x RIPA lysis buffer (Pierce). Co-immunoprecipitation was performed on approximately 1mg of protein from each sample using 10 µg of anti-flag antibody. The protein complexes were analyzed on a 6% TBE non-denaturing polyacrylamide gel with a PageRuler Plus pre-stained ladder (Thermo scientific) as marker. Gels were fixed in 50% methanol/distilled water and stained with coomassie blue (GelCode Blue Stain, Pierce) at room temperature for 1 hour. The Coommassie Blue stained gel bands underwent tryptic digestion to extract the peptides for MS analysis [21]. Briefly, each digested peptide sample was loaded on an Agilent 1200 nano-capillary HPLC system (Agilent Technologies) with a 10 cm integrated µRPLC-electrospray ionization (ESI) emitter columns (made in-house), coupled online with a LTQ XP mass spectrometer (Thermo Fisher Scientific) for µRPLC-MS/MS analysis. Peptides were eluted using a linear gradient of 2% mobile phase B (acetonitrile with 0.1% formiac acid) to 42% mobile phase B within 40 min at a constant flow rate of 0.25 µL/min. The seven most intense molecular ions in the MS scan were sequentially selected for collision-induced dissociation (CID) using a normalized collision energy of 35%. The mass spectra were acquired at the mass range of *m/z* 350–1800. The ion source capillary voltage and temperature were set at 1.7 kV and 200°C, respectively. The MS/MS data were searched against p53 protein database using SEQUEST. The cut-off for legitimate identifications were: charge state dependent cross correlation (X_corr_) ≥2.0 for [M+H]^1+^, ≥2.5 for [M+2H]^2+^ and ≥3.0 for [M+3H]^3+^.

### DNA Methylation Microarray

DNA from fresh frozen GBM tumor specimens were used for hybridization on Infinium Human Methylation 450 BeadChip, following the Illumina Infinium HD Methylation protocol and published methods [22]. Annotation of the human *JMJD3* locus The UCSC human genome browser NCBI36/Hg18. Release date March 2006 (http://genome.ucsc.edu/).

### Methylation Specific Sequencing

Methylation analysis of the intragenic regulatory area of *JMJD3* was performed on genomic DNA extracted from GSC 827 treated 5 µm 5-Azacytidine for 4 days and from untreated control GSC 827. Bisulfite conversion of DNA samples was performed using EpiTect Bisulfite Kit (Qiagen), following manufacturer’s instructions. For amplification of the CpG islands, the following primers where used: 3′*JMJD3*_F:TTTGTTTTTTATTAATTTGTGTTTTT 3′ *JMJD3*_R: TCCTACAACTAAAACTTCCACCTAC. PCR products where then subcloned using the TOPO TA Cloning Kit (Invitrogen, Carlsbad, CA) following manufacturer’s instructions. PCR verified 24–32 clones where then sequenced using the vectors T3 priming sites.

### *JMJD3* Locus Annotation

*JMJD3* genomic annotation was done using the The UCSC human genome browser NCB136/Hg18 at (www.genome.ucsc.edu). The *JMJD3* regulatory domain was annotated using Open Regulatory Annotation Consortium (www.oreganno.org/oregano).

### Intracranial Tumor Cell Injection into SCID Mice

A plasmid, hg01508s1, containing the human *JMJD3* cDNA (KIAA0346) was obtained from the cDNA bank section of Kazusa DNA Research Institute, Japan. The *JMJD3* open reading frame (ORF) was PCR-amplified and cloned into a pLenti4/TO/V5-DEST Gateway vector (Invitrogen). Viruses were generated and purified using Invitrogen’s viraPower lentiviral expression system. An intracranial orthotropic model was utilized for evaluation of gliobastoma stem cell tumorgenicity as previously described [20]. Briefly, empty vector and JMJD3 infected GSC 827 were dissociated and resuspended in 2 µl of HBSS. 1E^5^ GSCs, as determined by trypan blue dye exclusion, were injected stereotactically into the lateral ventricles of neonatal SCID mice. This study was carried out in strict accordance with the recommendations from the National Cancer Institute Animal Care and Use Committee who approved the study. Animals were euthanized by carbon dioxide inhalation. All efforts were made to minimize suffering.

## Results

### *JMJD3* is Induced during Differentiation of GSCs

**Figure 1:**
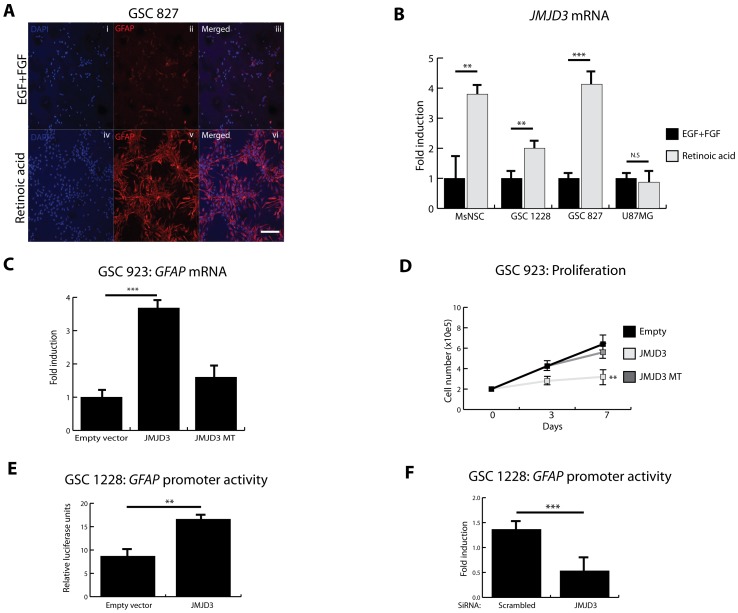
*JMJD3* is induced during differentiation of GSCs. A, Immunofluorescence of glioblastoma stem cells 827 (GSC 827) showing expression of mature astrocyte marker glial fibrillary acid protein (GFAP, *in red*) in proliferative media consisting of epidermal growth factor (EGF) and fibroblast growth factor (FGF) (**i–iii**) and differentiation media with 2 µM all-trans retinoic acid (RA) (**iv–vi**). DAPI (blue) depicts nuclei. Scale bar represents 20 µm. **B,** Quantitative real-time PCR (RT-qPCR) showing effect of retinoic acid (RA) on *JMJD3* transcriptional activity during differentiation of mouse neural stem cells (MsNSC), glioblastoma stem cells (GSCs) and serum grown U87 malignant glioblastoma cell line (U87MG). **C,** RT-qPCR on GSC 923 (wild-type *INK4A/ARF*, wild-type *TP53*) showing the effect of wild-type JMJD3 and mutant JMJD3 (MT) overexpression on *GFAP* expression. **D,** Effect of JMJD3 and JMJD3 MT overexpression on proliferation of GSC 923. **E–F,** The effect of *JMJD3* overexpression **(E; Luciferase assay)** and knockdown on *GFAP* promoter activity **(F; RT-qPCR) in GSC 1228 (** ***INK4A/ARF*** **null and wild type** ***TP53*** **)**. All experiments were done in triplicates. Error bars represent means ±SD. p = two-tailed Student’s *t* test comparing indicated samples, *p<0.1, **p<0.05, ***p<0.01, N.S.- not significant.

It has been shown that retinoic acid (RA) induces partial cell cycle arrest and differentiation of primary glioblastoma stem cells (GSCs) thereby inhibiting their tumorigenic potential [14], [23]. Consistent with these reports, RA promoted glial differentiation of GSC 827 as demonstrated by induction of glial fibrillary acid protein (*GFAP*) and astrocyte-like morphologic change (Figure 1A). As seen in normal mouse neural stem cells (MsNSCs) [4], we find that RA induces *JMJD3* in GSCs but not in the long-established U87MG glioma cell line (Figure 1B). This suggests that induction of *JMJD3* may not occur in more differentiated cell types but only in cells with stem cell-like properties. We investigated this possibility by comparing RA induction of *JMJD3* in the CD15/SSEA-1^+^ subpopulation of primary GSC 827 based on our prior report demonstrating that CD15/SSEA-1^+^ enriches for a population of tumor cells with stem cell-like properties [13]. We purified CD15/SSEA-1^+^ and CD15/SSEA-1^−^ from GSC 827 by magnetic activated cell sorting (MACS) (Table S1) and treated with RA. RA induced *JMJD3* and *GFAP* in the CD15/SSEA-1^+^ subpopulation, whereas RA did not cause induction of *JMJD3* or differentiation in the CD15/SSEA-1^−^ population (Figure S1). These data suggest that JMJD3 may be involved in the induction of differentiation programs in tumor stem cells in much the same way it activates such programs in normal stem cells.

### *JMJD3* Modulates Differentiation of GSCs Independently of *INK4A/ARF* Locus

During oncogene-induced senescence, JMJD3 demethylates H3K27me3 relieving repression of the *INK4A/ARF* promoter. This results in p16/p14 expression, p53 protein stabilization and p21 induction leading to senescence of human and mouse fibroblasts [2], [24]. In GSC 923 (wild-type *INK4A/ARF*, wild-type *TP53;* Table S2), overexpression of wild-type JMJD3 (Intact catalytic domain. Figure S2) induced *GFAP* expression, while mutant JMJD3 (MT; Deleted catalytic domain. Figure S2) did not (Figure 1C). JMJD3 also significantly inhibited proliferation of GSC 923 consistent with its pro-differentiation effects (Figure 1D). Surprisingly, JMJD3 overexpression in *INK4A/ARF* null (but wild-type *TP53*) GSC 1228 also induced *GFAP* promoter activity (Figure 1E), while siRNA-mediated knockdown of *JMJD3* inhibited *GFAP* promoter activity (Figure 1F). These results suggest that JMJD3 may modulate differentiation of GSCs independently of its reported cell cycle associated chromatin target, *INK4A/ARF*.

**Figure 2:**
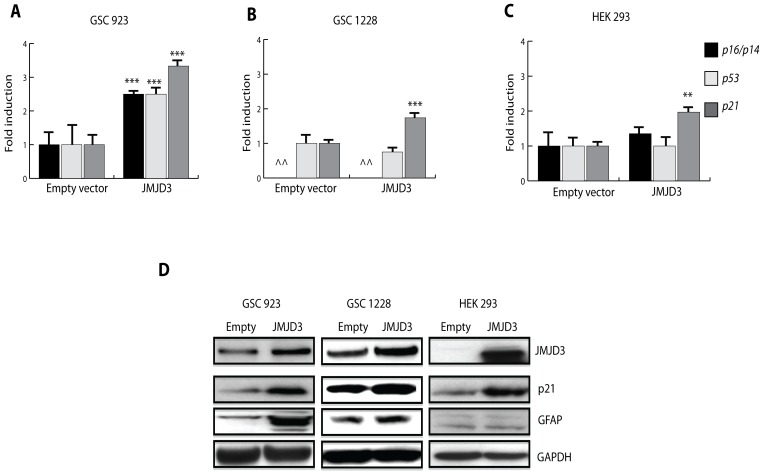
JMJD3 induces p21 in GSCs. A–C, Effect of JMJD3 overexpression on *p16/p14, p53 and p21* mRNA expression in GSC 923 (wild-type *INK4A/ARF* and *TP53*) (**A**) and GSC 1228 (^∧∧^ *INK4A/ARF* null, wild-type *TP53*) (**B**) and HEK 293 cells (wild-type *INK4A/ARF* and *TP53*) (**C**). **D**, Western blot analysis showing the effect of JMJD3 on p21 and GFAP protein expression in primary GSCs and HEK 293 cells. All experiments were done in triplicates. Error bars represent means ±SD. p = two-tailed Student’s *t* test comparing indicated samples to empty vector control, *p<0.1, **p<0.05, ***p<0.01, N.S.-not significant.

In wild-type *INK4A/ARF* and wild-type *TP53* GSC 923, JMJD3 induced *p16/p14* and *p21* mRNA expression (Figure 2A) consistent with re-activation of the *INK4A/ARF* locus via chromatin remodeling as shown in other cell types^6,7^. In *INK4A/ARF* null GSC 1228 and wild-type *INK4A/ARF* HEK 293 cells (both with wild-type *TP53*), however, JMJD3 also induced *p21* mRNA but without *p16/p14* or *p53* induction (Figure 2B, C) suggesting that JMJD3 mediated p21 induction in these cell types may be independent of both *INK4A/ARF* and *TP53* transcriptional activation. We also find that *p21* is induced during RA and serum mediated differentiation of GSCs (Figure S3), while exogenous p21 overexpression in GSCs is associated with *GFAP* expression (Figure S3). Moreover, JMJD3 mediated p21 protein expression is associated with GFAP protein induction in GSCs only (Figure 2D). Therefore, our results suggest that induction of *JMJD3* during differentiation of GSCs may promote cell cycle arrest and partial glial differentiation through p21 activation, independently of *INK4A/ARF* or *TP53* transcriptional control.

### JMJD3 Induction of p21 is p53-dependent

**Figure 3:**
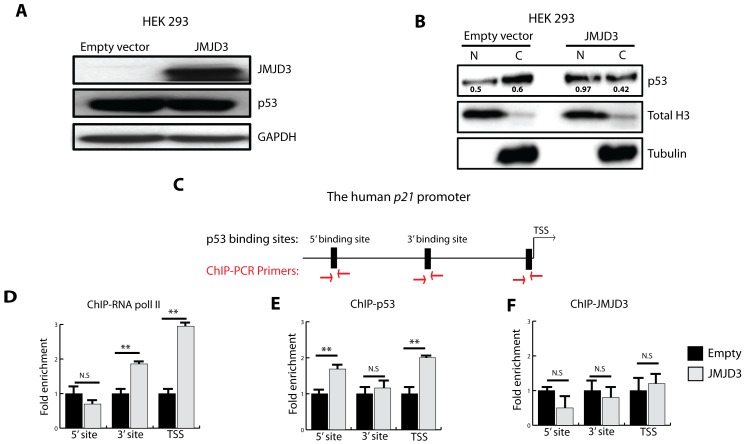
*JMJD3* induction of p21 is p53-dependent. **A,** Western blot analysis showing the effect of JMJD3 overexpression on total p53 levels in HEK 293 cells. **B,** Nuclear fractionation following JMJD3 overexpression in HEK 293 cells (N-Nuclear fraction, C-Cytoplasmic fraction). Total Histone 3 (Total H3) was used as nuclear loading control and Tubulin as cytoplasm loading control. Densitometry values of p53 expression (black) are relative to respective nuclear or cytoplasmic control. **C,** Schema of human *p21* promoter showing p53 consensus binding sites and primers used for Chromatin immunoprecipitation with PCR (ChIP-PCR). TSS is transcription start site, 3′ and 5′ sites represent consensus p53 binding sites within the human *p21* promoter. **D–F,** ChIP-PCR results showing localization of RNA polymerase II (**D**), p53 (**E**) and JMJD3 (**F**) at the *p21* promoter in HEK 293 cells following JMJD3 overexpression. All experiments were done in triplicates. Error bars represent means ±SD. p = two-tailed Student’s *t* test comparing indicated samples to empty vector controls, *p<0.1, **p<0.05, ***p<0.01, N.S.- not significant.

**Figure 4:**
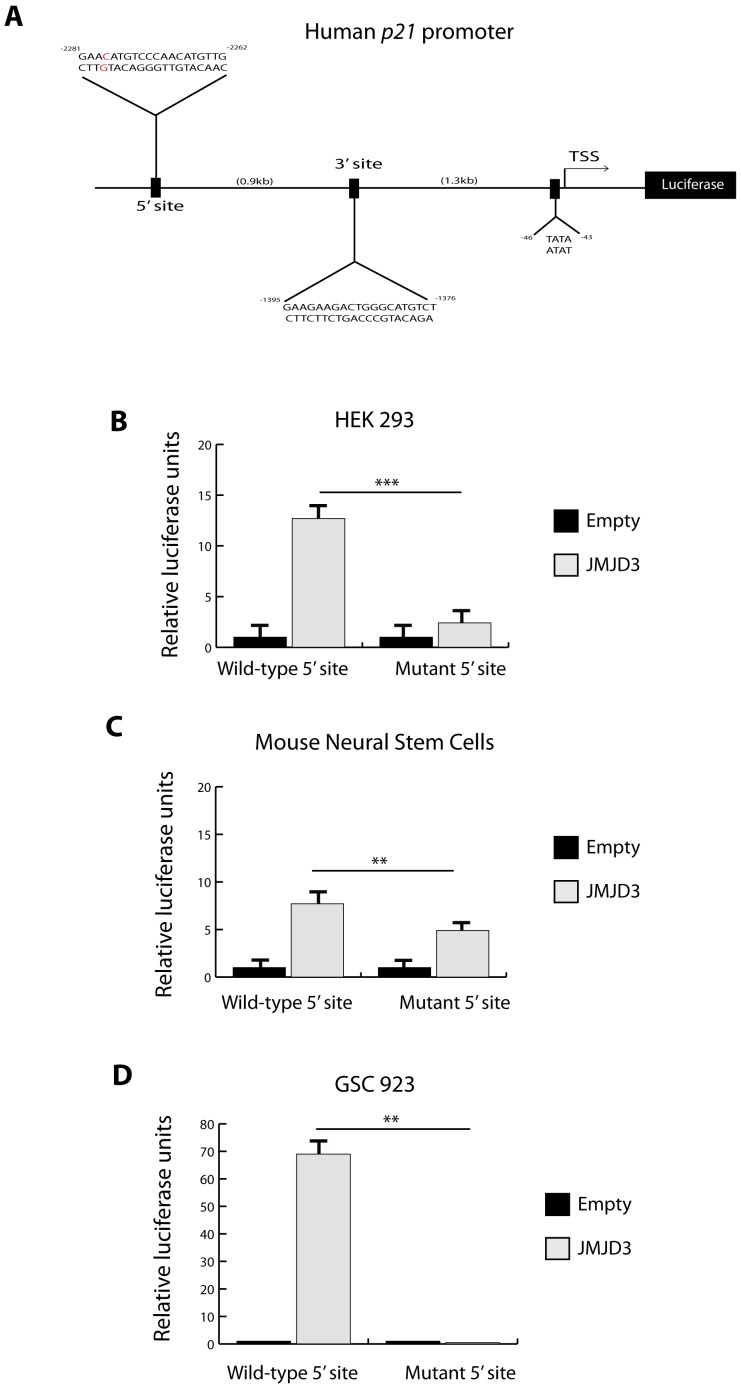
Effect of JMJD3 on the human *p21* promoter. A–D, Effect of JMJD3 overexpression on human *p21* promoter luciferase reporter (**A**) in HEK 293 (**B**), mouse neural stem cells E14 (MsNSCs) (**C**) and GSC 923 (**D**) with a wild-type p53 5′ binding site (wild-type 5′ site) and mutant p53 5′ binding site (mutant 5′ site; Position 2279 C>A *in red* (***A***)). TSS is transcription start site, 3′ and 5′ sites represent consensus p53 binding sites within the human *p21* promoter. All experiments were done in triplicates. Error bars represent means ±SD. p = two-tailed Student’s *t* test comparing indicated samples, *p<0.1, **p<0.05, ***p<0.01, N.S.-not significant.

We next investigated how JMJD3 could activate p21 independently of transcriptional up-regulation of p14 (*ARF*) or p53 (*TP53)*. JMJD3 overexpression in HEK 293 cells did not affect total p53 levels (consistent with a lack of *INK4A/ARF* or *TP53* transcriptional activation) but promoted its nuclear accumulation (Figure 3A, B). ChIP analysis of the endogenous human *p21* promoter (Figure 3C) shows that JMJD3 overexpression results in accumulation of RNA polymerase II (Figure 3D) and p53 (Figure 3E), but not JMJD3 at the *p21* promoter (Figure 3F). Moreover, mutation of the p53 binding site on an episomal non-chromatin based human *p21* promoter luciferase reporter significantly diminished *p21* promoter activation by JMJD3 in HEK 293, mouse neural stem cells and GSC 923 (Figure 4A–D). These data indicate that increased p53 nuclear localization and subsequent accumulation at the *p21* promoter mediates chromatin independent JMJD3 induction of p21.

**Figure 5:**
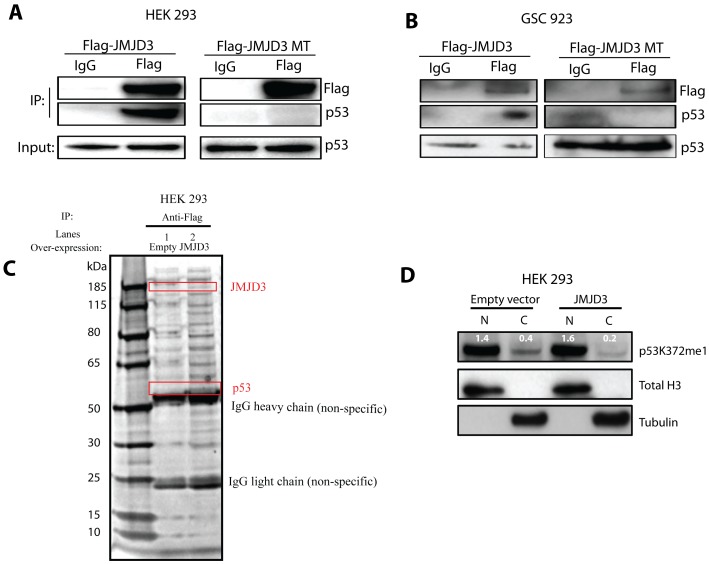
JMJD3 stabilizes p53 protein. A–B, Co-immunoprecipitation in HEK 293 cells (**A**) and GSC 923 (**B**) following overexpression of wild-type JMJD3 (in-tact catalytic domain) and mutant JMJD3 (MT; deleted catalytic domain) and western blot analysis for endogenous p53 protein. IP, immunoprecipitation. **C,** Mass spectrometry on JMJD3 interacting proteins in HEK 293 cells. **D,** Effect of JMJD3 overexpression on the cellular distribution of mono-methylated p53 lysine 372 (p53K372me1). N-Nuclear fraction and C-Cytoplasmic fraction. Total Histone 3 (Total H3) was used as nuclear fraction control and Tubulin as cytoplasmic fraction control. Densitometry values of p53K372me1 (white) are relative to respective nuclear or cytoplasmic control.

We then explored a potential non-chromatin modifying mechanism responsible for JMJD3-mediated p53 pathway activation. Recent evidence suggests that histone lysine modifiers may target non-histone substrates. For example, histone methyltransferases G9a, SET7 and SET9 induce the methylation of many non-histone substrates [25] including p53. Additionally, the H3K4me3 demethylase LSD1 was shown to demethylate lysine 370 on the p53 c-terminus, resulting in repression of p53 stability and function [26]. Co-immunoprecipitation experiments demonstrate that JMJD3 interacts with endogenous p53 in both HEK 293 (Figure 5A) and GSC 923 (Figure 5B; confirmed by liquid chromatography/mass spectrometry (LC/MS); Figure 5C). Further studies revealed that the c-terminal jumonji domain (JmjC) of JMJD3 is required for this interaction as a JmjC-deletion mutant JMJD3 does not interact with p53. Given its role as a histone lysine demethylase, we asked if JMJD3 induces p21 via direct lysine demethylation of p53. A previous publication demonstrated that the interaction between JMJD3 and p53 results in demethylation of lysine residues on p53 [27]. Specific lysine residues, however, were not assessed or characterized as potential JMJD3 targets on p53. Thus, given the impact of JMJD3 over-expression on differentiation in *INK4A/ARF* null but wild-type *TP53* GSCs, we sought to characterize specific lysine targets of JMJD3 on p53 via methylation profiling with mass spectrometry (MS). MS, however, was unable to detect baseline methylation on p53 protein in control samples. Next, we increased protein amounts as well as enriched for p53 protein from nuclear and cytoplasmic extracts through immunoprecipitation, however, baseline methylation on p53 protein could not be established. We were, therefore, unable to confirm or repudiate previous published evidence of lysine demethylation following JMJD3-p53 interaction [27].

Given a global decrease in lysine methylation on p53 following JMJD3 interaction [27], we looked for putative methylated lysines on p53, that following modification not only promote the stability of p53 but also activate the p21 promoter. It was previously reported that mono-methyl p53 lysine 372 (p53K372me1) is an active form of p53 previously shown to mediate p21 activation [28]. Here, we find that JMJD3 overexpression promoted nuclear retention of a p53K372me1, more than doubling the ratio of nuclear to cytoplasmic p53K372me1 (Figure 5D). Given that our MS experiments did not provide direct evidence of active p53 demethylation, the mechanism for enhanced nuclear localization of p53K372me1 remains unresolved. Therefore, we propose that p53 lysine 372 demethylation may be one of several mechanisms contributing to p53 nuclear retention during JMJD3-mediated differentiation of glioblastoma stem cells.

### *JMJD3* (*KDM6B)* Locus Harbor Aberrations in GBM

To assess the potential relevance of JMJD3 in human gliomagenesis, we looked for naturally occurring alterations within the *JMJD3* locus that could affect *JMJD3* activity or expression in GBMs. First, we queried whole exome sequence data from The Cancer Genome Atlas (TCGA) [29] as well as sequence data from primary GSCs derived in the Neuro-Oncology Branch at the National Institutes of Health. We found at least 3 different missense mutations and insertions within the TCGA database, (Table S3). Likewise, 3 of our 5 whole exome sequenced GSCs: GSC 308, GSC 1228 and GSC 827, also harbor missense mutations within the *JMJD3* locus (Table S3).

**Figure 6:**
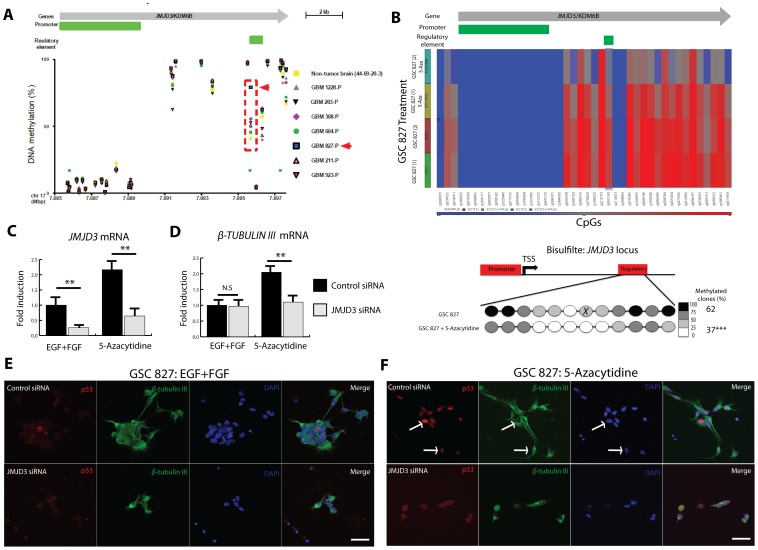
Regulatory element DNA demethylation re-activates *JMJD3* mRNA in GSC 827. A, *Illumina Infinium Human Methylation 450 bead chip* methylation arrays on parental GBM tumor samples (GBM-P) compared to non-tumor brain tissue (Non-tumor brain 44-69-20-3) showing the methylation status (0%-unmethylated, 100%-hypermethylated) of *JMJD3* regulatory regions (Chromosome 17. UCSC NCBI 36/Hg 18). **B,** (*Top)* Heat map from whole genome DNA methylation profiling showing that 5-Azacytidine reproducibly demethylates the *JMJD3* regulatory element (from 2 independent experiments). (*Bottom)* Bisulfite sequencing confirmed *JMJD3* regulatory element in GSC 827 demethylation following 5-Azacytidine treatment. No significant changes were found at the promoter. *X* indicates cg09911083. **C–D,** Effect of 5-Azacytidine on *JMJD3* mRNA (**C**) expression as well as differentiation markers *β*-*TUBULIN* III (*TuJ1*) (**D**) in GSC 827. *JMJD3 SMARTpool* siRNA knockdown inhibits 5-Azacytidine induced differentiation of GSC 827. **E–F,** Immunocytochemistry of GSC 827 showing the effects of *JMJD3* knockdown on p53 nuclear accumulation before (**E**) and after (**F**) 5-Azacytidine treatment as assessed by morphology and β-TUBULIN III expression. *Arrows pointing to differentiated GSCs with nuclear p53.* Scale bar represents 20 µm. All experiments were done in triplicates. Error bars represent means ±SD. p = two-tailed Student’s *t* test comparing indicated samples to EGF+FGF control, *p<0.1, **p<0.05, ***p<0.01, N.S.- not significant.

To look for epigenetic changes in primary GBMs that could affect JMJD3 expression and function, whole genome DNA methylation arrays were performed on a series of parental glioblastoma samples (GBM-P, n = 7) which demonstrated that compared to non-tumor brain tissue, all GBM samples show relative hypermethylation of a CpG site (Illumina cg09911083) within the *JMJD3* intragenic regulatory element (Figure 6A; Chr.17p13.1∶7,695,644–7,696,309; UCSC NCBI36/Hg18[30]–[32]). By contrast, the *JMJD3* promoter shows no significant hypermethylation in non-tumor brain tissue (Figure 6A). For example, primary GBM 827 (GBM 827-P) demonstrates highly significant levels of methylation at the *JMJD3* regulatory element relative to non-tumor brain tissue and other tumor samples (Figure 6A; red arrow). *JMJD3* expression is also relatively suppressed in GBM 827-P compared to non-tumor brain and other tumors GBM 206-P and GBM 923-P (Figure S4) while western blot analysis showed lower JMJD3 protein expression in GSC 827 compared to other GSCs (Figure S4). These results suggest that methylation of the *JMJD3* regulatory element may affect JMJD3 expression in GBM 827.

### JMJD3 Re-activation Induces a p53-mediated Differentiation of GSCs

The publically available UCSC genome browser contains ChiP-sequencing data and whole genome methylation profiles from several cell lines including embryonic stem cells and cancer cell lines. [30]–[33]. By analyzing data within the UCSC browser genome browser, we found that cg019911083 is part of a CpG island (Chromosome 17p13.1∶7,695,644–7,696,309. UCSC NCBI36/Hg18) previously annotated as a ‘positive regulatory element’ [34], [35] (Open Regulatory Annotation OREG0024145. Chromosome 17p13.1∶7,695,692–7,696,642. UCSC NCBI36/Hg18)[30]–[33]. This region also demonstrates enhancer-like features based on published regulatory element properties [30], [36] such as evolutionary conservation, DNase hypersensitivity, enhancer-promoter associated histone mark (H3K4me1), RNA polymerase II occupancy and binding by transcription factors (TF) reported to activate *JMJD3* expression such as NF-κB [3], [30]–[33] and the retinoic acid receptor RXR-A[4], [30]–[33]. Therefore, to determine if hypermethylation of this intragenic *JMJD3* enhancer-like region, is a regulatory mechanism contributing to suppression of *JMJD3* expression in gliomas such as GBM 827-P, we treated GSC 827 with 5-Azacytidine and confirmed *JMJD3* regulatory element (but not promoter) demethylation through bisulfite sequencing (Figure 6B). Regulatory element demethylation re-activates *JMJD3* mRNA expression (Figure 6C) and induces differentiation markers *β*-*TUBULIN* III (Figure 6D) and *GFAP* (Figure S5) in GSC 827. Furthermore, siRNA mediated knockdown of *JMJD3* following DNA demethylation in GSC 827 inhibited this differentiation (Figure 6D, Figure S5 and Figure S6 for protein knockdown confirmation). These results suggest that DNA hypermethylation of the *JMJD3* regulatory element negatively regulates *JMJD3* transcription and differentiation of GSC 827.

Next, we assessed if RA mediated *JMJD3* induction in GBM GSCs is regulatory element methylation-dependent. We find that RA treatment of GBM 827 GSCs induces *JMJD3* without changes to the methylation status of the JMJD3 promoter or downstream regulatory element (Data not shown) suggesting that RA induces *JMJD3* via a methylation independent mechanism. In normal neural stem cells (NSCs), it was shown that the RA nuclear co-repressor SMRT/NCOR-2 controls the differentiation of neural stem cells into neurons [4] and astrocytes [11]. In GSC 827, we find that RA mediated astrocytic differentiation is associated with a decrease in nuclear SMRT/NCOR-2 protein, JMJD3 induction and p53 nuclear accumulation (Figure S7). These findings suggest that JMJD3 mediated p53 pathway activation may be suppressed in GBMs via multiple mechanisms.

Wild-type JMJD3 over-expression has been previously shown to promotes p53 nuclear accumulation in normal NSCs [27]. To establish whether regulatory element mediated *JMJD3* re-activation induces differentiation of GSCs via p53 protein activation, independently of *INK4A/ARF,* we assessed p53 nuclear localization following 5-azacytidine treatment of *INK4A/ARF* null (but wild-type *TP53*) GSC 827. 5-Azacytidine significantly induced p53 nuclear accumulation with morphological evidence of differentiation in GSC 827 (Figure 6E–F, Figure S8). SiRNA mediated knockdown of *JMJD3* following 5-azacytidine treatment of GSC 827, however, significantly inhibited p53 nuclear accumulation and morphologic evidence of differentiation (Figure 6F and Figure S8). These results demonstrate that JMJD3 mediates p53-induced differentiation of GSCs independently of the *INK4A/ARF* locus.

### Relevance of *JMJD3* Expression *in vivo*

**Figure 7:**
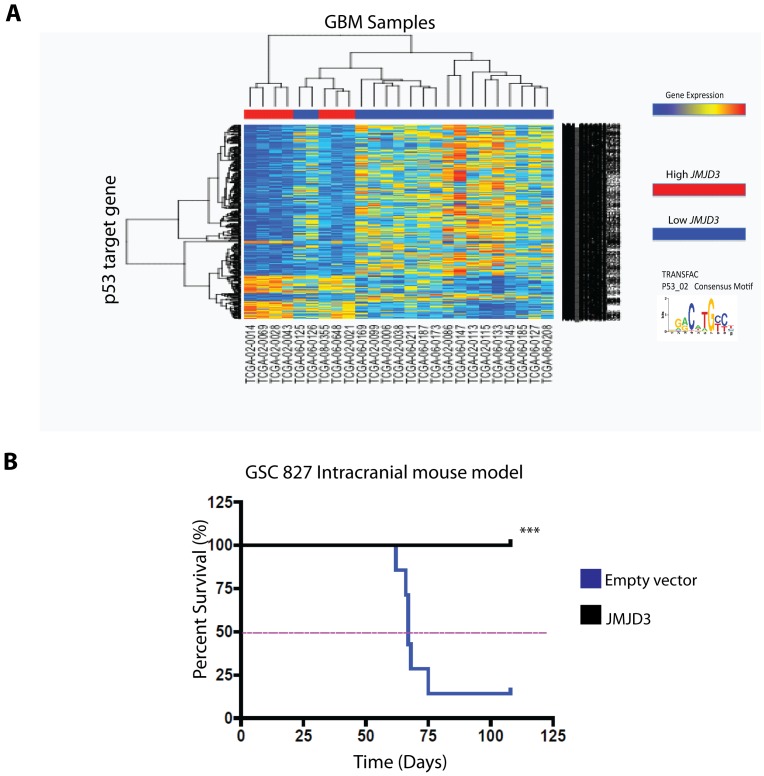
Relevance of JMJD3 expression *in* ** ***vivo.*** **A,** 672 differentially expressed p53 motif associated genes from TCGA GBM samples (wild-type *TP53*) with high *JMJD3* (Fold change >1.5; n = 7) and low *JMJD3* (Fold change <1; n = 18) expression. These genes contain a DNA motif in the -450/+50 promoter region that scores 85% of total maximum possible for the p53 motif (TRANSFAC P53_02 motif). **B**, JMJD3 overexpression suppresses tumorigenicity of GSC 827 *in vivo* (***Log Rank P = 0.0029).

To determine if the above *in vitro* effects of JMJD3 on p53 have any clinical relevance, we asked whether *JMJD3* expression in parental GBM samples with wild-type *TP53* influences p53 activity within those samples. To investigate this hypothesis, we examined upstream promoter regions (−450/+50 of the Transcription Start Site) of genes differentially expressed between GBM *TP53* WT samples with high *JMJD3* expression versus low *JMJD3* expression. Using the PSCAN motif enrichment algorithm, we found that a consensus binding motif for p53 is significantly enriched in these promoters (fdr < = .05) greater than would be expected by chance. This set of differentially expressed genes showed predominant repression of p53 target genes in high *JMJD3* expressing GBMs compared with low *JMJD3* expressing GBMs (Figure 7A). These data are consistent with previous reports that demonstrate that following p53 activation as many as 80% of p53 responsive genes are repressed rather than activated [37], [38] and suggest that the p53 pathway is more active in high *JMJD3* expressing GBMs retaining wild-type *TP53* than in GBMs with wild-type *TP53* but low *JMJD3* expression. Consistent with these findings, mice with intracranial stereotactically injected JMJD3 over-expressing GSC 827 cells lived dramatically longer than mice injected intracranially with parental GSC 827 cells (Figure 7B) further demonstrating the tumor suppressive effects of JMJD3 *in vivo*.

**Figure 8:**
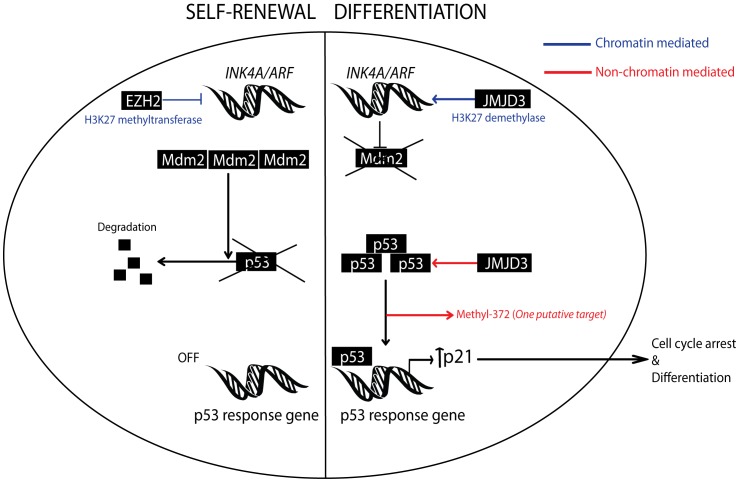
Schematic model. Showing how JMJD3 activation of the p53 pathway in stem cells via chromatin and non-chromatin dependent mechanisms contributes to differentiation. It also demonstrates that EZH2 over-expression as seen in some GBMs may promote self-renewal of stem cells by suppressing the p53 pathway.

## Discussion

There is increasing evidence that a small subpopulation of cells within human glioblastomas (GBMs) is instilled with tumor-initiating and neural stem cell properties (glioma stem cells, or GSCs). The fact that GSCs can be driven toward terminal differentiation *in vitro*, but rarely *in vivo* suggests, at least in part, epigenetic regulation of differentiation. Consistent with this supposition, we previously reported that a small subset of GBMs had GSCs that were temporally and developmentally fixed in a pro-proliferative, non-differentiated state through epigenetic silencing of the bone morphogenic protein receptor 1b [20]. More recently, it was demonstrated that IDH1 mutations promoted aberrant histone demethylase activity resulting in a histone methylation (H3K9me3 and H3K27me3) mediated block to differentiation [1], [16]. This was in part due to the inhibition of α-ketoglutarate (α-KG) dependent enzymes such as the H3K9me3 demethylase, KDM4C. Amongst the many unresolved questions, however, is the relevance of H3K27me3 demethylases in GBMs and cancer in general.

In normal neural stem cells, the H3K27 specific demethylase JMJD3 was shown to modulate differentiation by affecting p53 nuclear distribution [27]. The implications of these findings in any cancer model, however, remains unclear. Here, we reveal that the H3K27-specific demethylase, JMJD3, promotes differentiation and suppresses proliferation of GSCs, not only through its well-known chromatin-dependent activation of the *INK4A/ARF* locus, but also through p53 protein nuclear stabilization. Based on previous studies demonstrating global lysine demethylation on p53 following interaction with JMJD3 in differentiating neural stem cells [27], our results suggest that lysine 372 modifications may represent one of many putative mechanisms resulting in p53 protein stabilization following interaction with JMJD3 resulting in differentiation and suppression of proliferation in glioblastoma stem cells. We also demonstrate that primary-patient-derived GSCs and primary human GBMs counter the tumor suppressor effects of JMJD3 through hypermethylation of an evolutionarily conserved intragenic and enhancer-like DNA regulatory element within the *JMJD3* locus or direct somatic mutations of the *JMJD3* gene. Pharmacological demethylation of GSC with hypermethylation-silenced JMJD3 or overexpression of a wildtype JMJD3 in *JMJD3* mutated GSCs results in p53 pathway activation and GSC differentiation *in vitro* and hugely extended survival of animals with intracranial GSC xenografts. The clinical relevance of these findings is reflected by our demonstration through genome wide transcriptional motif analyses in a large number of GBM patients that GBMs with high levels of *JMJD3* expression and wildtype *TP53* show significantly enhanced p53 pathway activation.

Furthermore, our findings describe an alternative mechanism (apart from 2-HG and α-KG) for how a DNA hypermethylation phenotype in tumors like GBMs may functionally modulate the activity/expression of the histone demethylases such as JMJD3 in tumors like GBM 827. Moreover, we speculate that DNA methylation may also affect *JMJD3* mRNA processing via transcription independent mechanisms (no impact on total mRNA expression levels). It was demonstrated that intragenic DNA hypermethylation may regulate differential exon inclusion during mRNA elongation [39]. Here, DNA methylation impairs CTCF binding to exon 5 and its inclusion into full length CD45 mRNA. The *JMJD3* regulatory element spans exon 17, which encodes for part of the *JMJD3* catalytic domain called jumjonji C (JmjC; Exons 14–17), a region previously shown to be absolutely required for the demethylase activity of JMJD3 (22 exons) [40]. Whole genome ChIP-sequence data from embryonic stem cells in the UCSC genome browser online also show that CTCF binds the *JMJD3* regulatory element, [30]–[32]. Thus, it is possible that intragenic DNA hypermethylation could also impair JMJD3 activity by modulating CTCF-mediated JmjC demethylase domain inclusion into full length *JMJD3* mRNA. Therefore, apart from modulating the activity of methylation sensitive enhancers (as in GSC 827), intragenic hypermethylation in some GBMs could also contribute to suppression of JMJD3 enzymatic activity resulting in an aberrant block to terminal differentiation. If true, the aberrant methylation within the *JMJD3* loci may prove to be a useful therapeutic target in cancer.

Methylation independent mechanisms also exist for JMJD3 reactivation in GSCs. It has been shown that during neuronal differentiation SMRT/NCOR-2 directly inhibits JMJD3 transcriptional activation by promoter-mediated repression, resulting in a H3K27-mediated repression of differentiation genes [4]. RA however, relieves this block by inducing the destabilization of nuclear SMRT/NCOR-2 resulting in JMJD3 activation and differentiation of NSCs [4]. SMRT/NCOR-2 has also been previously shown to modulate the differentiation state and tumorigenicity of GSCs [23], [41]. Here, RA-mediated destabilization of nuclear SMRT/NCOR-2 [23] or serine/threonine protein phosphatase 2A (PP2A)-mediated inhibition [41] results in differentiation and suppresses proliferation of GSCs. Thus, JMJD3 may be re-activated in GSCs via methylation dependent and independent mechanisms.

Methylation and demethylation of p53 has been previously shown to modulate p53 stability and function [26]–[28], [42], [43]. Others have demonstrated that the interplay between methylation and acetylation of the p53 protein activates p53 during DNA damage response [44]. Also, methylation of specific lysines has been shown to affect the ability of p53 to interact with co-activators involved in p53-mediated apoptosis [26]. Therefore, it is speculated that the interplay between methylation, demethylation and acetylation may fine tune p53 activity during tumor initiation and progression [45]. It remains largely unclear, however, how methylation specifically stabilizes or destabilizes p53. To this end, following our results showing the significant impact of a lysine demethylase on p53 stability in cancer, it will be necessary to perform methylation profiling on p53 to definitively establish whether demethylases such as JMJD3 stabilize p53 through direct demethylation and if not, through what other mechanisms.

In conclusion, the relevance of most histone demethylases, including the H3K27me3 demethylase, JMJD3, in cancer remains unclear. Here, we propose that JMJD3 acts as a tumor suppressor gene in glioblastoma multiforme. We find that JMJD3 interacts with p53 protein in GSCs and JMJD3 re-activation in GSCs induces p53 nuclear accumulation in an *INK4A/ARF* null background resulting in a p53-mediated differentiation of GSCs (See model in figure 8). These findings point to a role for histone demethylases in cancer and suggest that JMJD3 may serve as a tumor suppressor gene and a potential therapeutic target in glioblastoma multiforme.

## Supporting Information

Figure S1 Retinoic acid induces *JMJD3* in CD15^+^/SSEA-1^+^ subpopulation only. **A,** Retinoic acid induces *JMJD3* in the CD15^+^/SSEA-1^+^ GSC 827 stem cell subpopulation only. **B,** Induction of *JMJD3* in CD15^+^/SSEA-1^+^ GSC 827 is associated with glial differentiation as measured by *GFAP* mRNA expression.
(JPG)

Figure S2 Wild-type JMJD3 demethylates H3K27me3. **A–B,** Validation of wild-type JMJD3 (intact catalytic domain) ability to demethylate H3K27me3 in HEK 293 cells **(A)**. Mutant JMJD3 (MT; deleted catalytic domain) does not affect H3K27me3 levels **(B)**. *Arrows showing cells of interest expressing either flag-tagged JMJD3 or JMJD3 MT.* Scale bar represents 20µm.
(JPG)

Figure S3 p21 contributes to differentiation of GSCs. **A,** Quantitative real time PCR (RT-qPCR) showing the effect of retinoic acid and serum on *p21* expression in GSC 923. **B–C,** RT-qPCR showing the effect of transient p21 overexpression **(B)** on *GFAP* expression **(C)** in GSC 923.
(JPG)

Figure S4 *JMJD3* expression in glioblastoma tumor samples. **A,** Quantitative real-time PCR (RT-qPCR) showing relative levels of *JMJD3* mRNA in non-tumor brain tissue and parental GBM samples (GBM-P). **B,** Western blot analysis showing JMJD3 protein expression across primary glioblastoma stem cells (GSCs). All experiments were done in triplicates. Error bars represent means ±SD. p = two-tailed Student’s *t* test comparing indicated samples to non-tumor brain tissue, *p<0.1, **p<0.05, ***p<0.01, N.S.-not significant.
(JPG)

Figure S5 JMJD3 knockdown inhibits differentiation of GSC 827. Effect of 5-Azacytidine on *GFAP* mRNA expression with and without JMJD3 knockdown. All experiments were done in triplicates. Error bars represent means ±SD. p = two-tailed Student’s *t* test comparing indicated samples, *p<0.1, **p<0.05, ***p<0.01, N.S., not significant.
(JPG)

Figure S6 Assessing JMJD3 siRNA knockdown efficiency in GSC 827. Showing JMJD3 siRNA efficiently suppresses 5-Azacytidine mediated JMJD3 protein induction in GSC 827. EGF+FGF is control growth factor media.
(JPG)

Figure S7 NCoR2 mediates repression of JMJD3 expression in GSC 827. Effect of RA on nuclear NCoR2 during differentiation of GSC 827. NCoR2 destablization is associated with JMJD3 induction and p53 nuclear accumulation. N-Nuclear fraction and C-Cytoplasmic fraction. Total Histone 3 (Total H3) was used as nuclear fraction control and Tubulin as cytoplasmic fraction control.
(JPG)

Figure S8 Effect of *JMJD3* knockdown on p53 nuclear accumulation in GSC 827. **A–B,** Immunocytochemistry showing effect of *JMJD3* knockdown on nuclear p53 and GFAP expression under proliferating conditions (EGF+FGF) **(A)** and following 5-Azacytidine **(B)**. *Arrow pointing to a differentiated GSC with nuclear p53.* Scale bar represents 20µm.
(JPG)

Table S1 Magnetic activated cell sorting of GSC 827. **A,** FACS analysis on unsorted GSC 827 showing the prevalence of CD15^+^/SSEA-1^+^ cells, the putative cancer stem cell population. **B,** FACS analysis on sorted fractions of GSCs showing prevalence of each sub-population following double sorting of GSC 827.
(JPG)

Table S2 Characteristics of glioblastoma samples used in the study. Showing genomic status of *INK4A/ARF* and *TP53* loci of glioblastoma samples used in the study.
(JPG)

Table S3 Whole exome sequence analysis from the *JMJD3* locus in human glioblastoma. Somatic mutations within the *JMJD3* locus in glioblastoma (GBM) from the Cancer Genome Atlas (TCGA) and the National Institutes of Health (NIH). SNV-Single nucleotide variation, Het- Heterozygous, Hom-Homozygous.
(JPG)
