# Supplementary figures and images for: Histone Demethylase Jumonji D3 (JMJD3) as a Tumor Suppressor by Regulating p53 Protein Nuclear Stabilization
Source: PLoS One. 2012 Dec 7;7(12):e51407. doi: 10.1371/journal.pone.0051407 (PMC3517524; doi:10.1371/journal.pone.0051407)

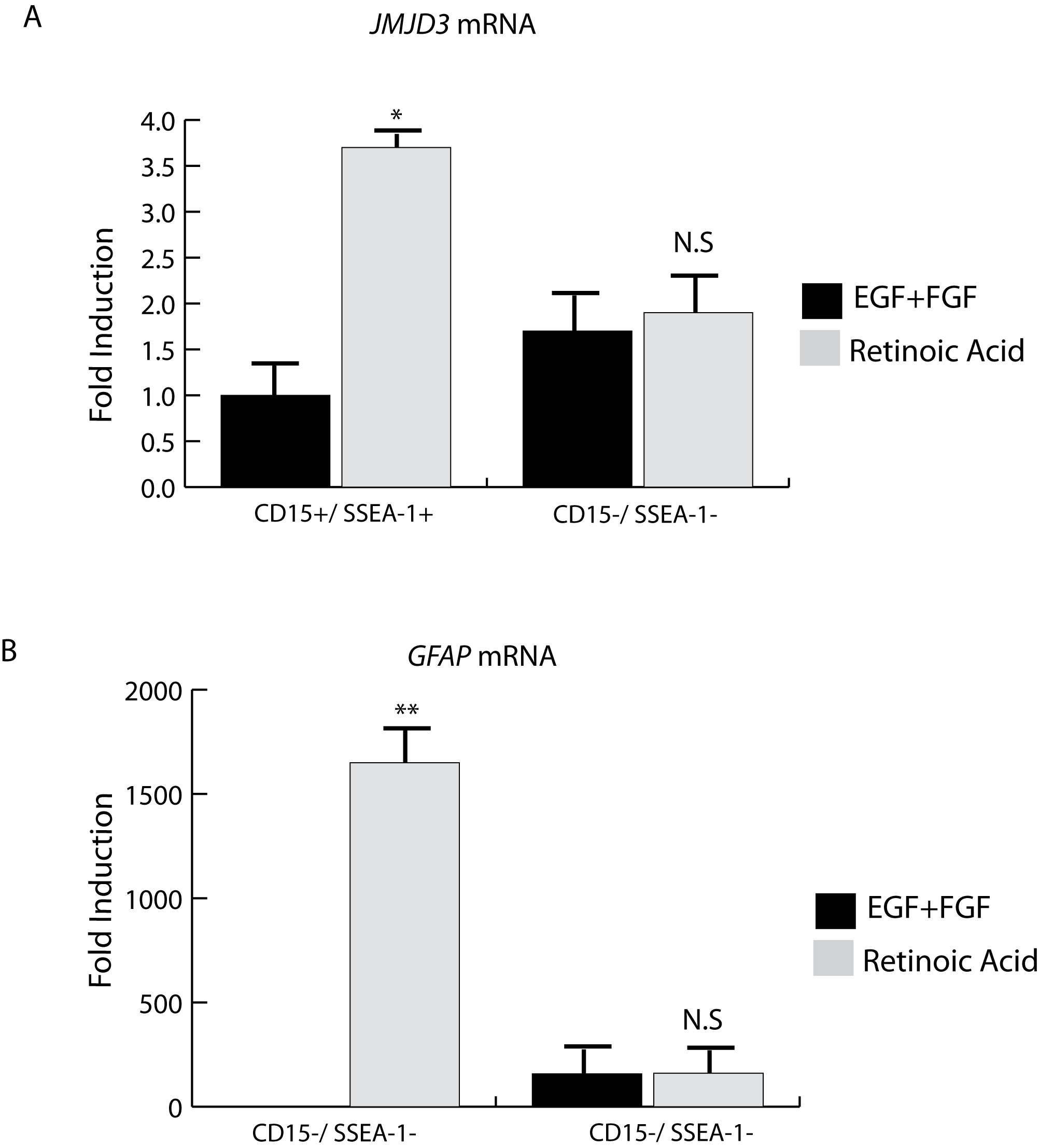

Supplement: Figure S1 — Retinoic acid induces JMJD3 in CD15+/SSEA-1+ subpopulation only. A, Retinoic acid induces JMJD3 in the CD15+/SSEA-1+ GSC 827 stem cell subpopulation only. B, Induction of JMJD3 in CD15+/SSEA-1+ GSC 827 is associated with glial differentiation as measured by GFAP mRNA expression. (JPG) [file pone.0051407.s001.jpg]

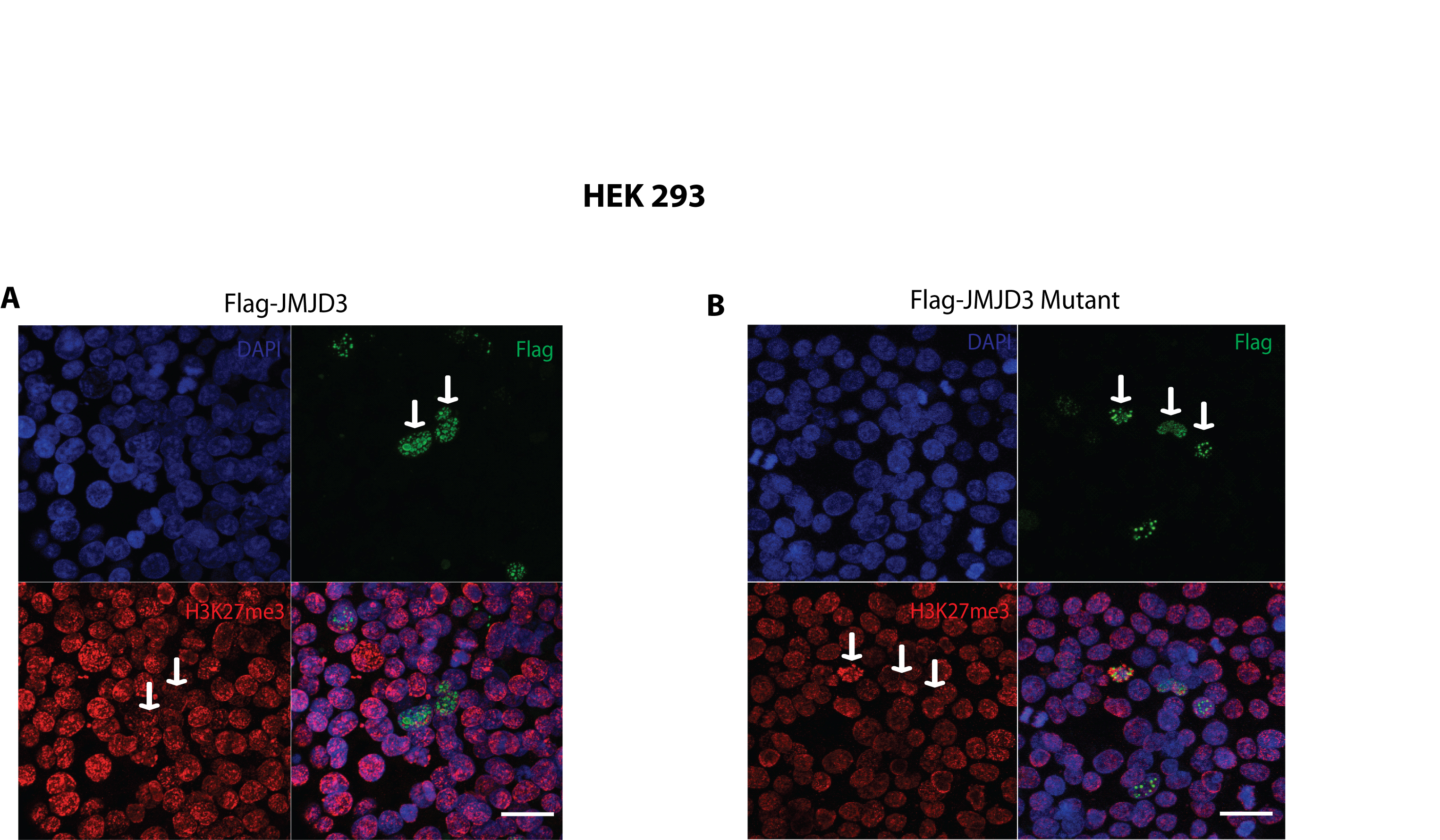

Supplement: Figure S2 — Wild-type JMJD3 demethylates H3K27me3. A–B, Validation of wild-type JMJD3 (intact catalytic domain) ability to demethylate H3K27me3 in HEK 293 cells (A). Mutant JMJD3 (MT; deleted catalytic domain) does not affect H3K27me3 levels (B). Arrows showing cells of interest expressing either flag-tagged JMJD3 or JMJD3 MT. Scale bar represents 20µm. (JPG) [file pone.0051407.s002.jpg]

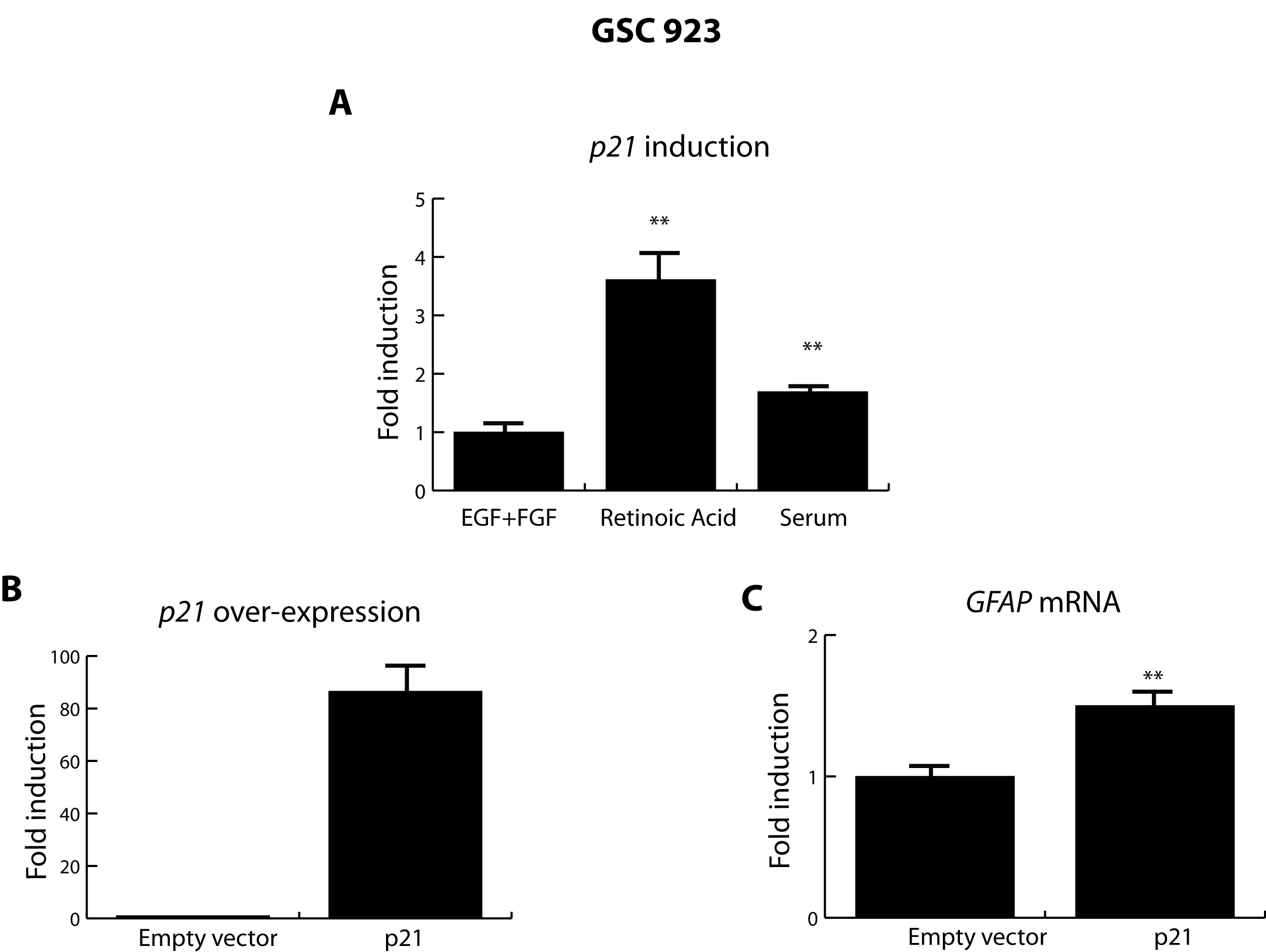

Supplement: Figure S3 — p21 contributes to differentiation of GSCs. A, Quantitative real time PCR (RT-qPCR) showing the effect of retinoic acid and serum on p21 expression in GSC 923. B–C, RT-qPCR showing the effect of transient p21 overexpression (B) on GFAP expression (C) in GSC 923. (JPG) [file pone.0051407.s003.jpg]

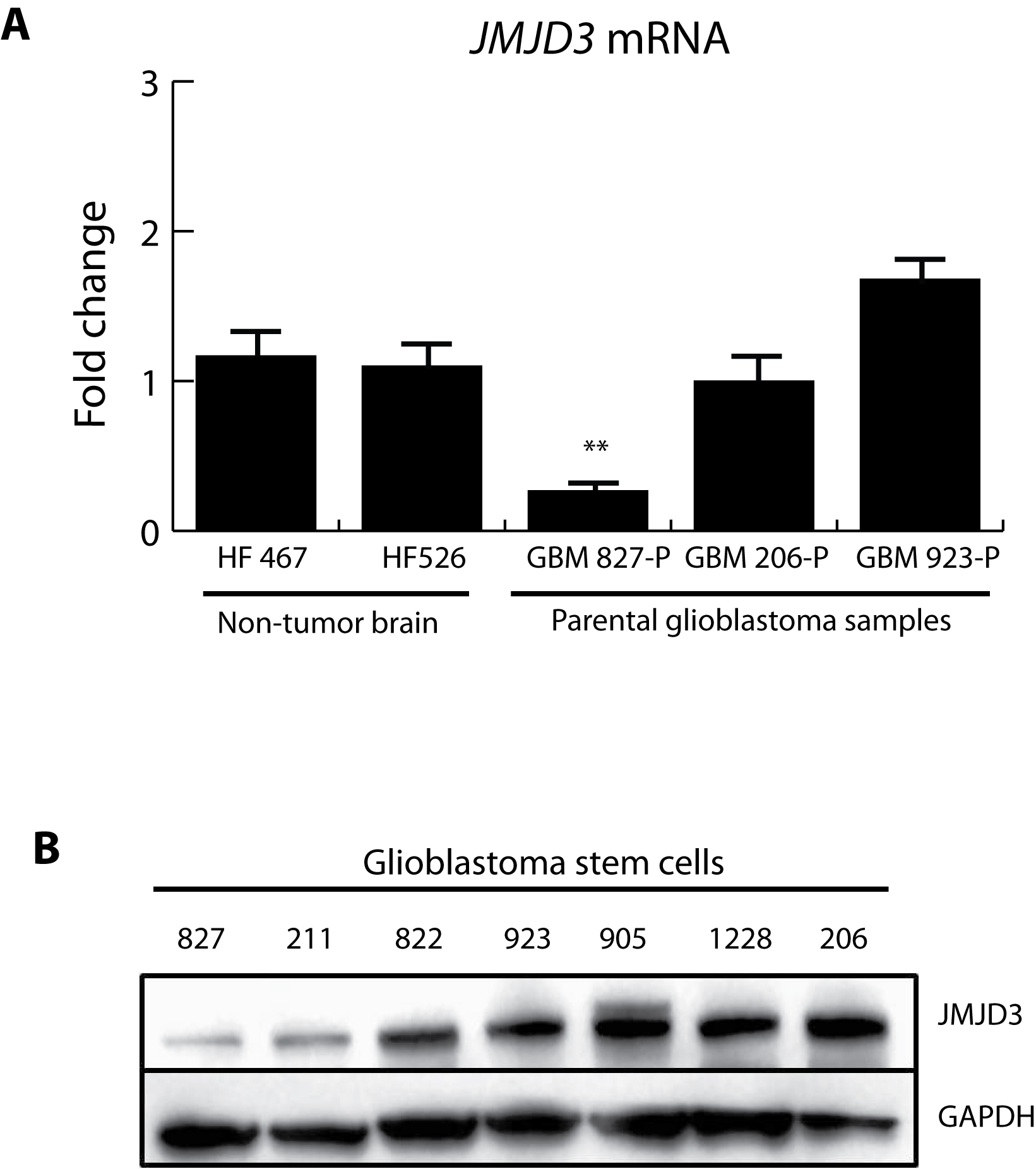

Supplement: Figure S4 — JMJD3 expression in glioblastoma tumor samples. A, Quantitative real-time PCR (RT-qPCR) showing relative levels of JMJD3 mRNA in non-tumor brain tissue and parental GBM samples (GBM-P). B, Western blot analysis showing JMJD3 protein expression across primary glioblastoma stem cells (GSCs). All experiments were done in triplicates. Error bars represent means ±SD. p = two-tailed Student’s t test comparing indicated samples to non-tumor brain tissue, *p<0.1, **p<0.05, ***p<0.01, N.S.-not significant. (JPG) [file pone.0051407.s004.jpg]

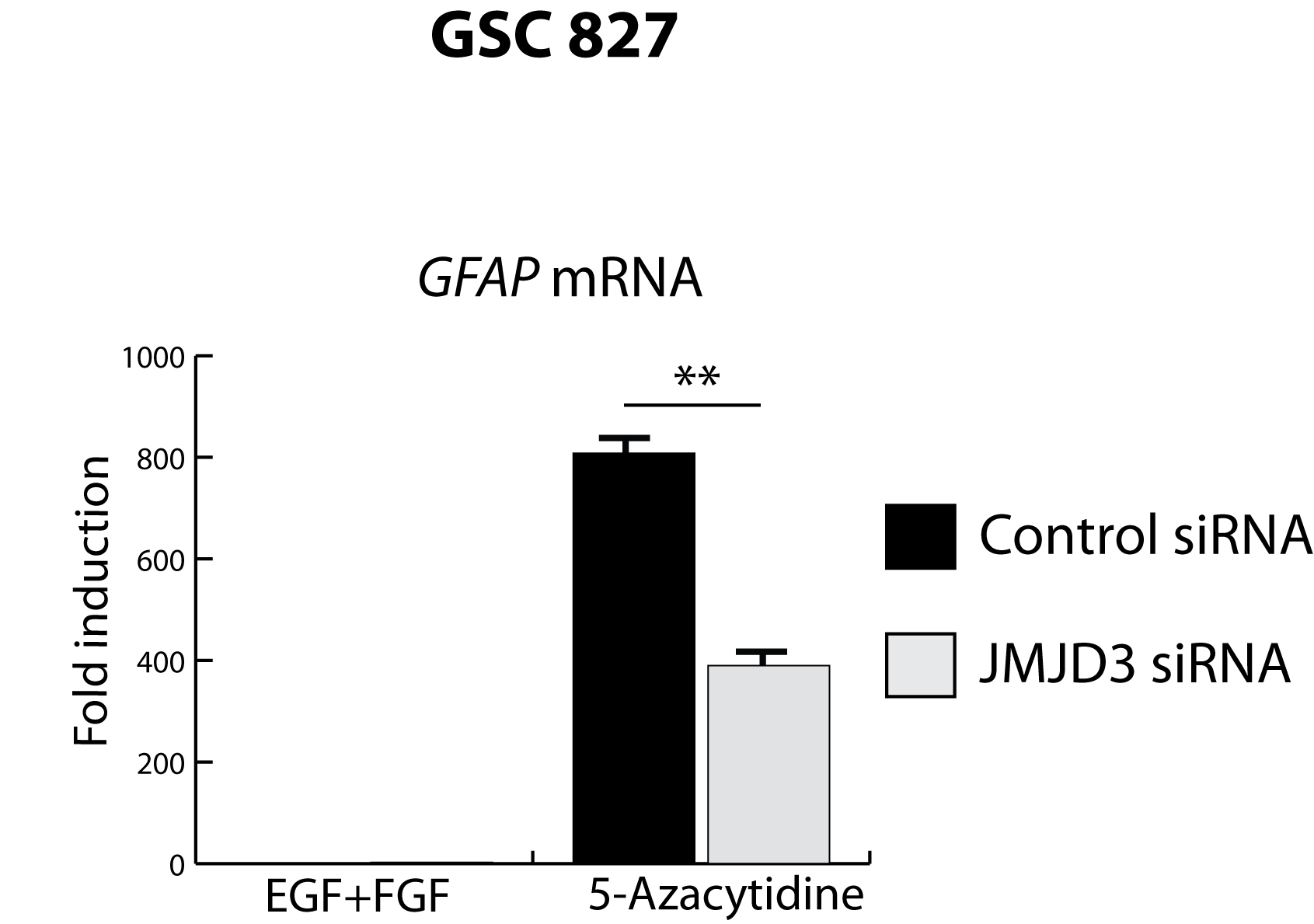

Supplement: Figure S5 — JMJD3 knockdown inhibits differentiation of GSC 827. Effect of 5-Azacytidine on GFAP mRNA expression with and without JMJD3 knockdown. All experiments were done in triplicates. Error bars represent means ±SD. p = two-tailed Student’s t test comparing indicated samples, *p<0.1, **p<0.05, ***p<0.01, N.S., not significant. (JPG) [file pone.0051407.s005.jpg]

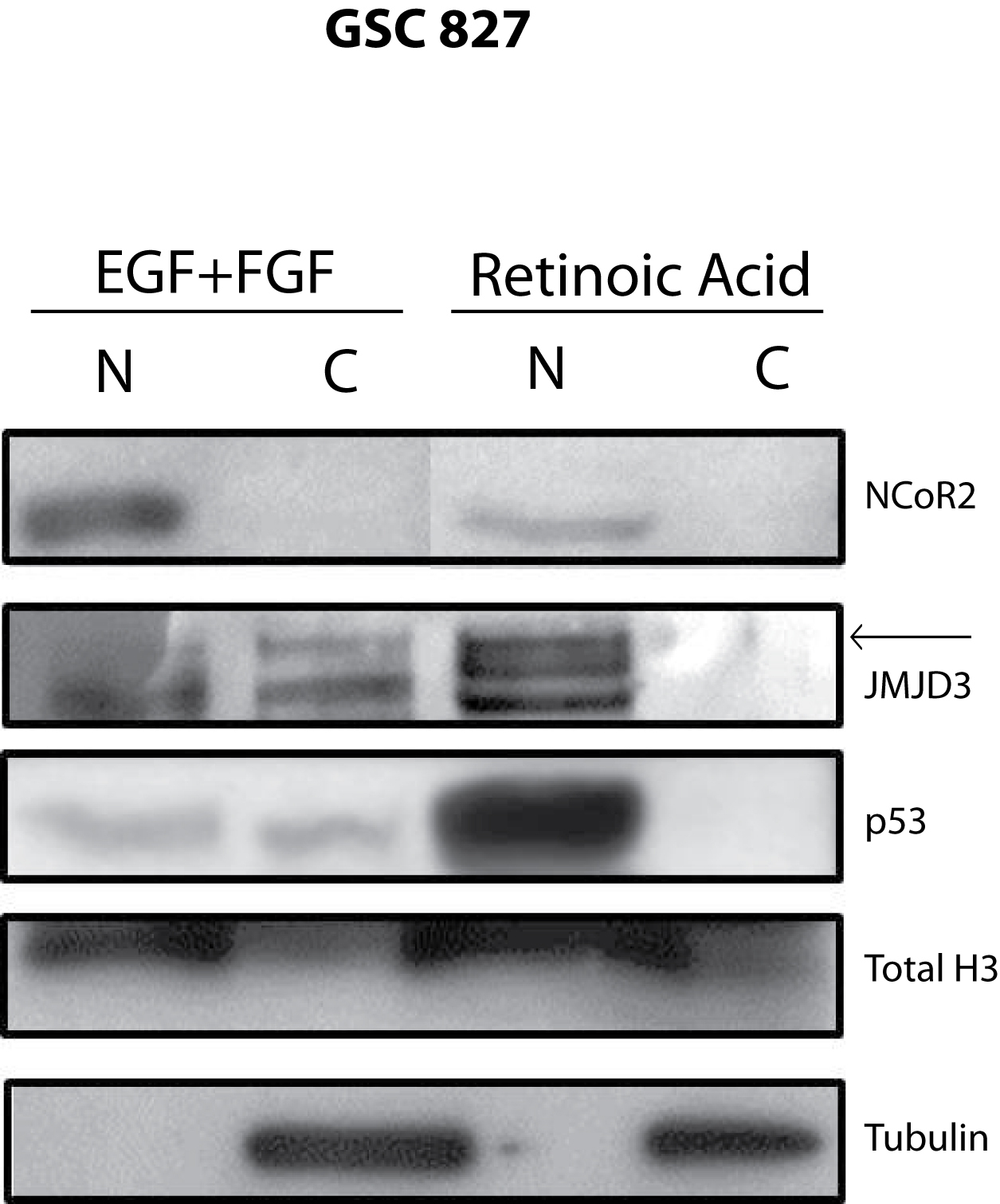

Supplement: Figure S6 — Assessing JMJD3 siRNA knockdown efficiency in GSC 827. Showing JMJD3 siRNA efficiently suppresses 5-Azacytidine mediated JMJD3 protein induction in GSC 827. EGF+FGF is control growth factor media. (JPG) [file pone.0051407.s006.jpg]

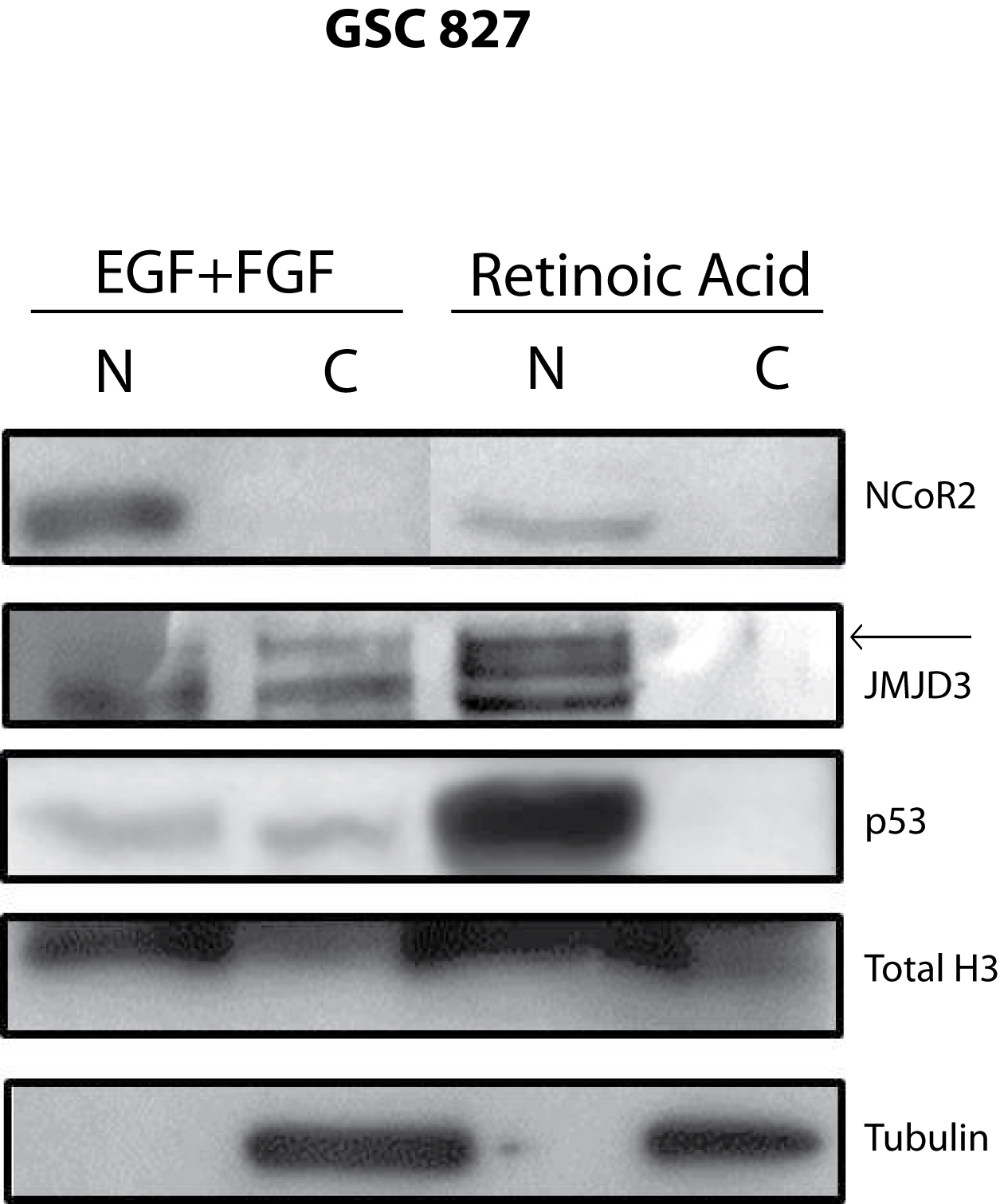

Supplement: Figure S7 — NCoR2 mediates repression of JMJD3 expression in GSC 827. Effect of RA on nuclear NCoR2 during differentiation of GSC 827. NCoR2 destablization is associated with JMJD3 induction and p53 nuclear accumulation. N-Nuclear fraction and C-Cytoplasmic fraction. Total Histone 3 (Total H3) was used as nuclear fraction control and Tubulin as cytoplasmic fraction control. (JPG) [file pone.0051407.s007.jpg]

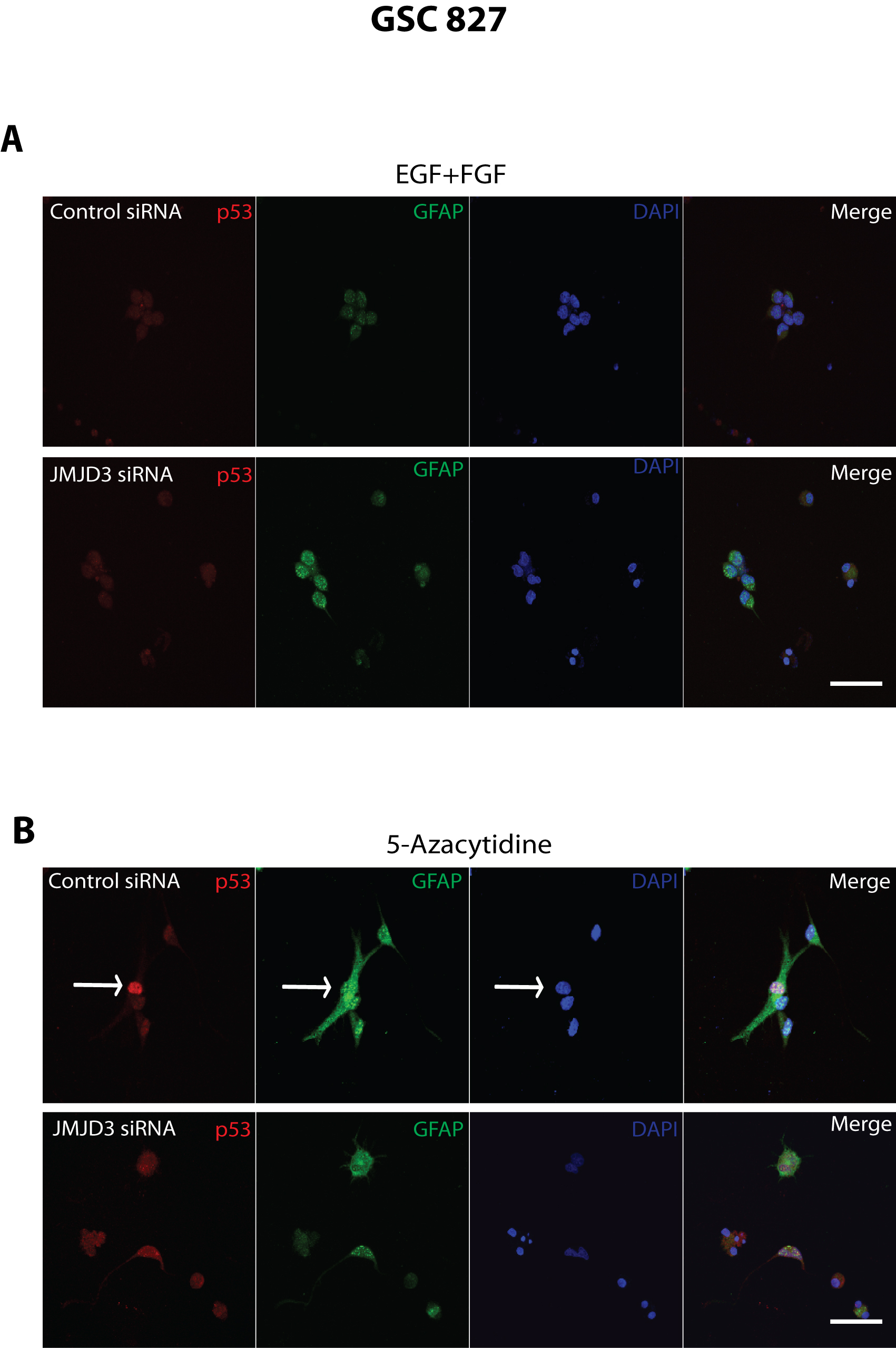

Supplement: Figure S8 — Effect of JMJD3 knockdown on p53 nuclear accumulation in GSC 827. A–B, Immunocytochemistry showing effect of JMJD3 knockdown on nuclear p53 and GFAP expression under proliferating conditions (EGF+FGF) (A) and following 5-Azacytidine (B). Arrow pointing to a differentiated GSC with nuclear p53. Scale bar represents 20µm. (JPG) [file pone.0051407.s008.jpg]

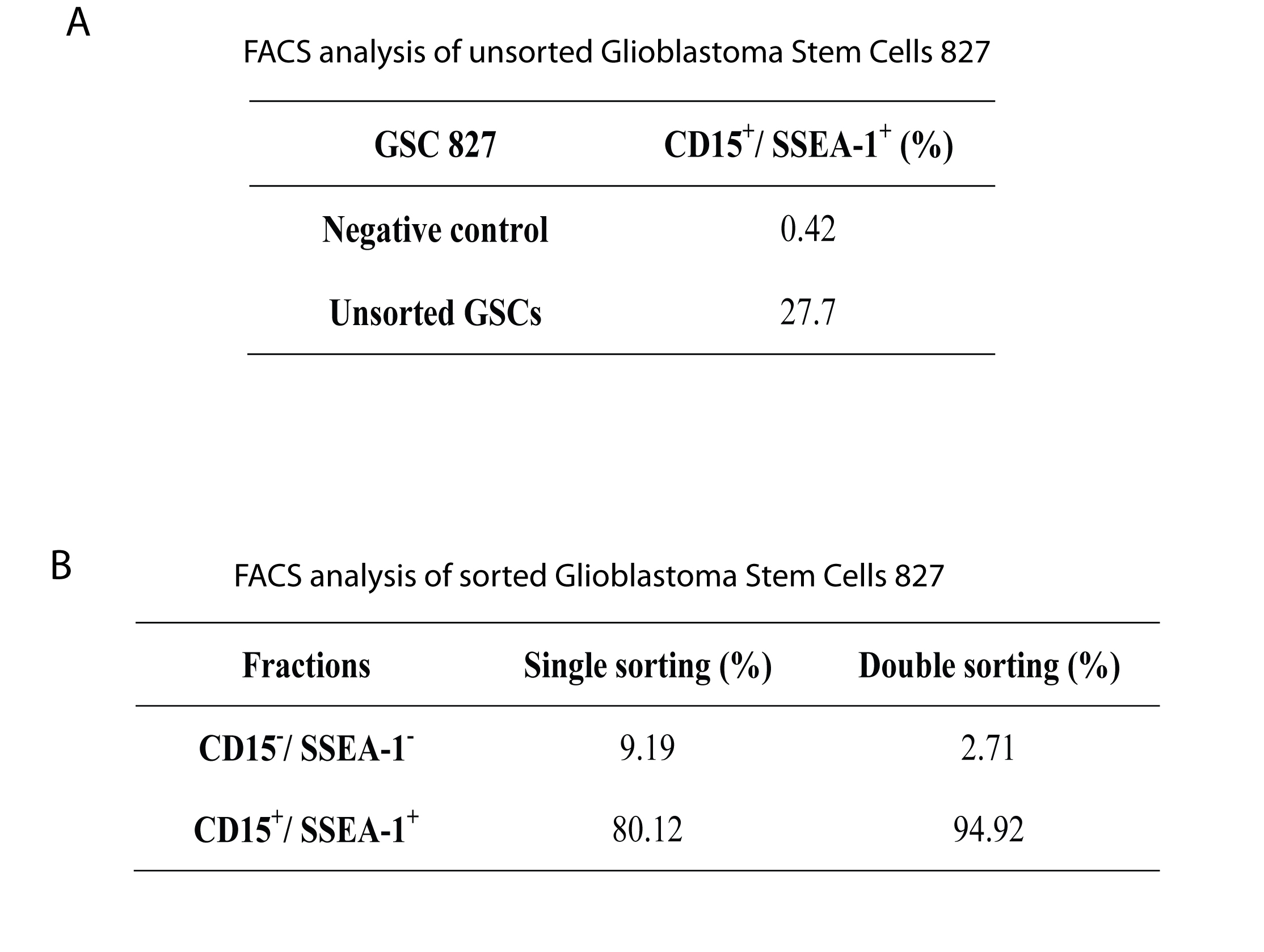

Supplement: Table S1 — Magnetic activated cell sorting of GSC 827. A, FACS analysis on unsorted GSC 827 showing the prevalence of CD15+/SSEA-1+ cells, the putative cancer stem cell population. B, FACS analysis on sorted fractions of GSCs showing prevalence of each sub-population following double sorting of GSC 827. (JPG) [file pone.0051407.s009.jpg]

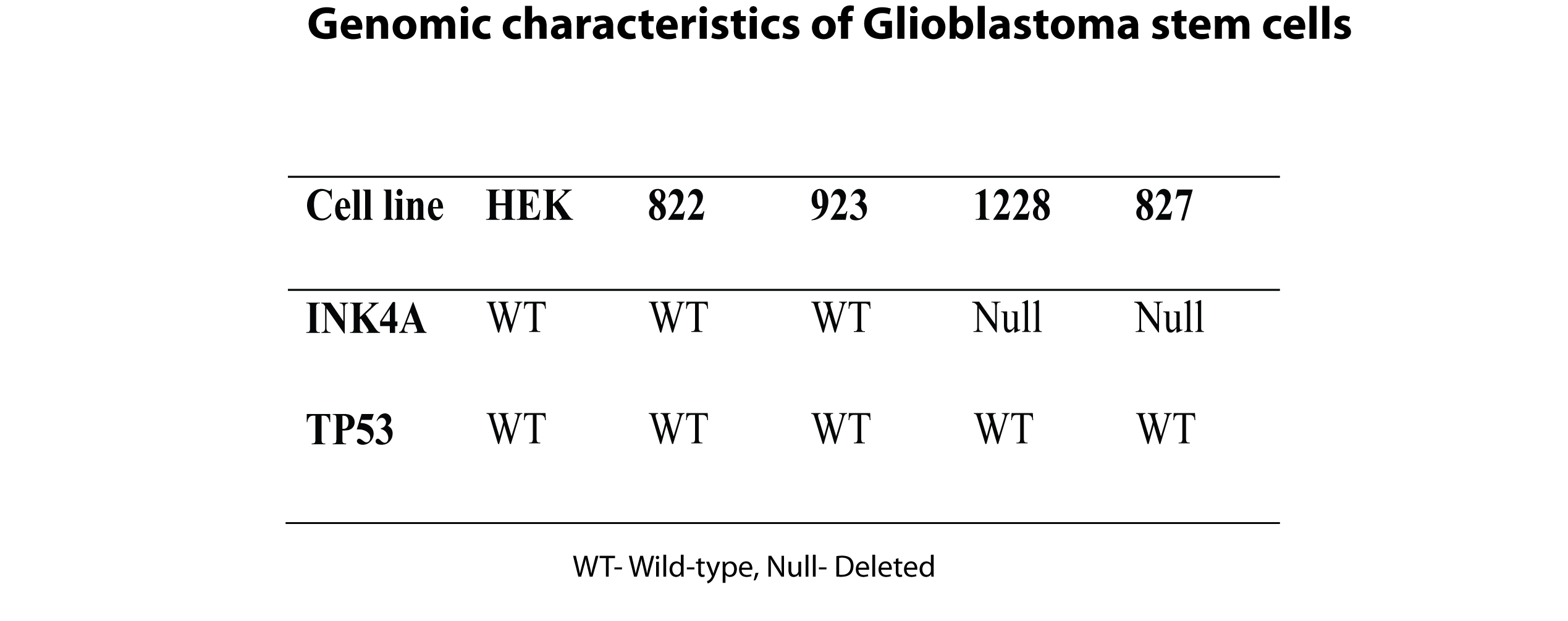

Supplement: Table S2 — Characteristics of glioblastoma samples used in the study. Showing genomic status of INK4A/ARF and TP53 loci of glioblastoma samples used in the study. (JPG) [file pone.0051407.s010.jpg]

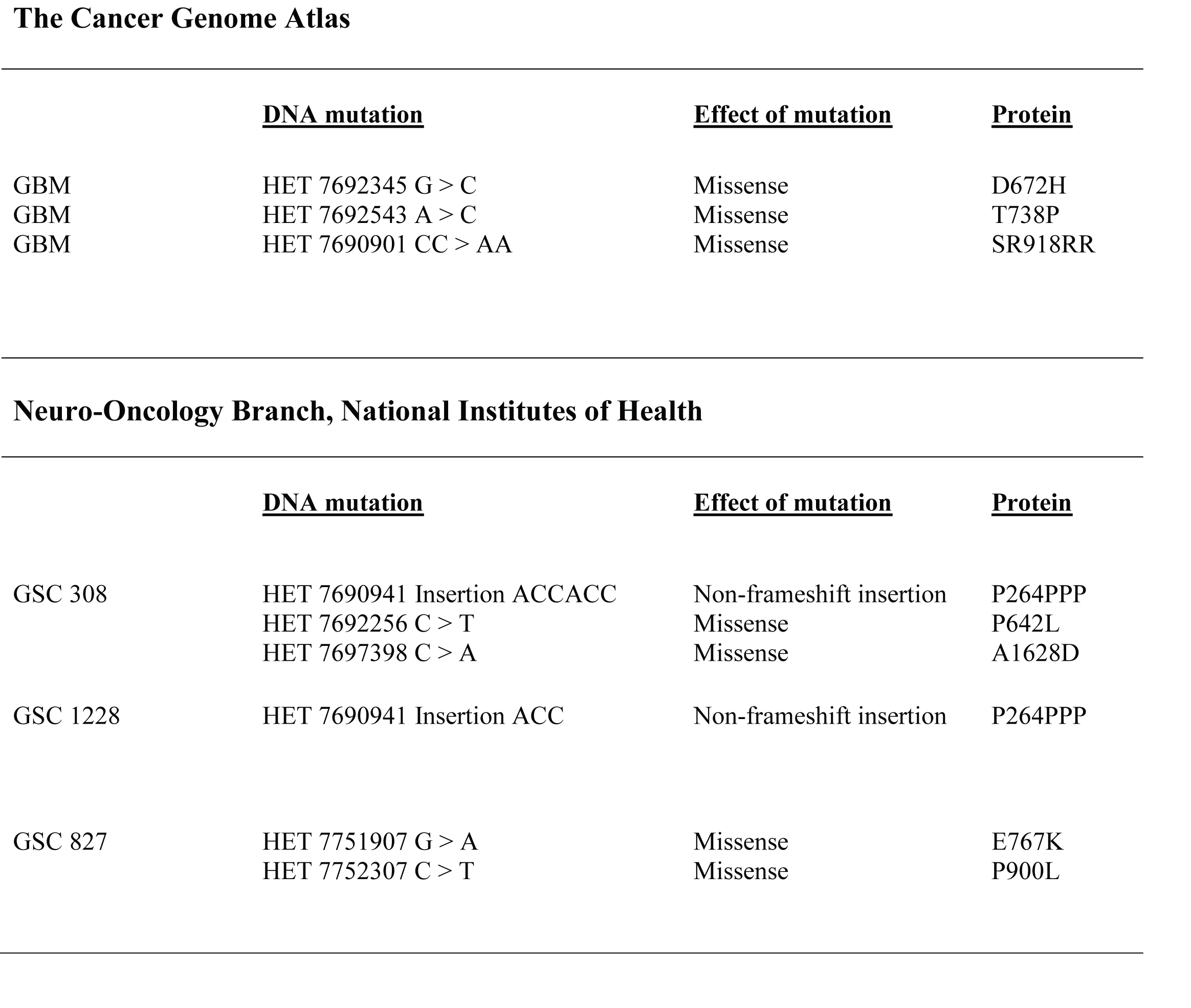

Supplement: Table S3 — Whole exome sequence analysis from the JMJD3 locus in human glioblastoma. Somatic mutations within the JMJD3 locus in glioblastoma (GBM) from the Cancer Genome Atlas (TCGA) and the National Institutes of Health (NIH). SNV-Single nucleotide variation, Het- Heterozygous, Hom-Homozygous. (JPG) [file pone.0051407.s011.jpg]
